# Assessing Short-Term Supply Disruption Impacts within Life Cycle Sustainability Assessment—A Case Study of Electric Vehicles

**DOI:** 10.1021/acs.est.3c05957

**Published:** 2023-11-13

**Authors:** Marcus Berr, Roland Hischier, Patrick Wäger

**Affiliations:** † Empa, Swiss Federal Laboratories for Materials Science and Technology, Lerchenfeldstrasse 5, 9014 St. Gallen, Switzerland

**Keywords:** Supply disruption impacts, Life cycle sustainability assessment, Criticality assessment, Supply chain, Electric vehicles, Cobalt, Aluminum, SPOTTER

## Abstract

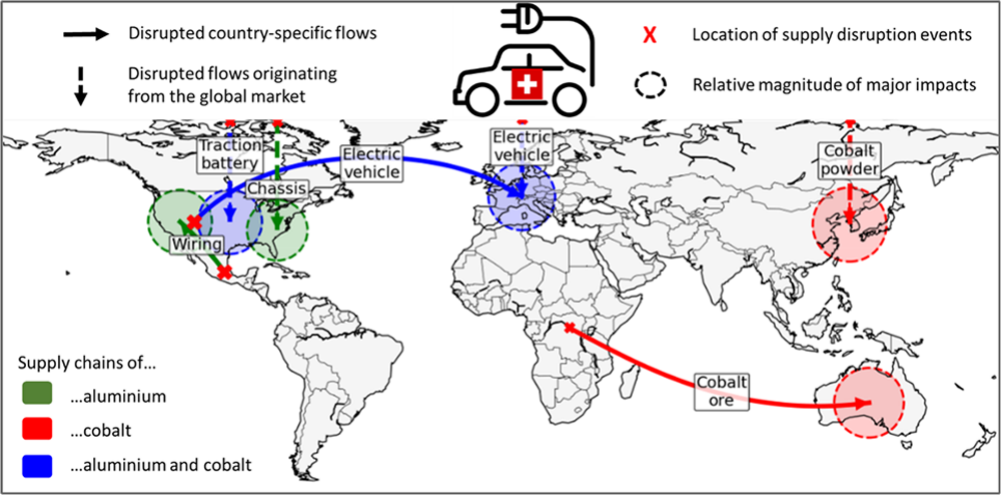

In this article, the recently published SPOTTER approach, which allows for identifying potential supply disruption impacts along the entire supply chain within life cycle sustainability assessment in the short term (i.e., < 5 years), is applied to a case study addressing the cobalt and aluminum supply chains of electric vehicles (EVs) used in Switzerland. Existing studies within the field assessing supply disruption impacts for EVs and other technologies focus on impacts related to raw material supply and thus neglect impacts along full supply chains. The present study identifies hotspots and overall impacts along the full supply chains by analyzing six supply disruption events (i.e., geopolitical instability, child labor restrictions, trade barriers, price volatility, limited recyclability, and economic resource depletion) for two impact categories (i.e., cost variability and limited availability). Identified hotspots suggest that supply chains are potentially disrupted mainly through events occurring in Asian, African, or other developing countries and affecting the Western economies. The highest risks are indicated in relation to the supply of EVs, EV wiring, traction batteries, cobalt powder, and cobalt ore. Suitable measures to mitigate these supply risks are suggested showing that some of the suggestions could not have been made based on the results of existing studies.

## Abstract 2

This research identifies supply disruption impacts and hotspots along entire supply chains of electric vehicles for Switzerland, playing an important role in reducing the country’s carbon emissions.

## 1 Introduction

Implementing electric vehicles (EVs) is a possibility to reduce carbon dioxide emissions in the mobility sectors in Switzerland and many other countries.^1−3^ However, events leading to disruptions of material and product flows along the supply chain of EVs may hinder their implementation and upscaling. Recently, the COVID-19 pandemic has caused shut-downs of traction battery production in China^4^ and global shortages of microprocessor chips in vehicles.^5^ Risks of further disruptions along EV supply chains are often estimated as high due to the complexity and fragility of the EV supply chain system^6^ and the dependency of EV manufacturing on so-called “critical raw materials” such as cobalt, lithium, or natural graphite.^7,8^ Cobalt for example is rated critical because, among other reasons, over 50% of its ore is mined in the Democratic Republic of the Congo,^9^ a country that is viewed as geopolitically unstable^10^ and where conflicts have frequently led to supply disruptions.^11^ More resilient supply chains and better risk management should be established for electromobility to reduce the supply risks.

Mechanisms leading to resilience in supply chains have been identified by Sprecher et al.^12,13^ using a case study from the 2010 rare earth crisis. These mechanisms include, for example, increases in supply diversity, improvements in material properties, and substitution. In another study, Sprecher and colleagues^14^ identify stockpiling as a suitable response option to supply disruptions caused by unexpected events for metals produced as coproducts.

To identify measures suitable for mitigating supply risks, potentially disrupted flows along supply chains first need to be anticipated. Here, criticality assessment is useful, as it allows for assessing the relative importance of supply disruptions for materials/products. Several critical studies have already been performed with regard to the electromobility sector. For example, Helbig et al.^15^ have used the criticality assessment approach developed by Tuma et al.^16^ to assess the supply disruption impacts for raw materials used for different traction batteries.

Other studies have assessed supply disruption impacts by applying criticality assessment approaches integrated into the life cycle sustainability assessment (LCSA) framework because such approaches offer, among other benefits, the possibility to avoid burden-shifting between supply disruption impacts and environmental impacts. For example, Gemechu et al.,^17^ Cimprich et al.,^18^ Santillan-Saldivar et al.,^19^ and Lütkehaus et al.^20^ have used and extended the GeoPolRisk approach developed by Gemechu et al.^21^ to evaluate the impacts of raw materials utilized in EVs or traction batteries. Henßler et al.^22^ in turn have applied the ESSENZ approach developed by Bach et al.^23^ to assess the impacts of metals and fuels used in plug-in hybrid electric vehicles.

While various approaches assessing criticality within LCSA have been developed (see a list of approaches in Cimprich et al.^24^ and Berr et al.^25^), these approaches mainly focus on raw material supply.^24,25^ There is thus a high risk of neglecting supply risks that must be mitigated in terms of creating resilient supply chains. To tackle this issue, Berr et al.^25^ have developed the SPOTTER approach that is assessing supply disruption impacts along the full supply chain within the LCSA framework.

The on-hand article aims at demonstrating the use of SPOTTER in a first case study, where impacts of supply disruptions are identified along the cobalt (Co) and aluminum (Al) supply chains of EVs used in Switzerland. EVs have been chosen as the case study object because of their growing importance as a more environmentally friendly mobility solution and the estimation of high disruption probabilities along their supply chains. Specifically, Co and Al supply chains are considered because Co and bauxite, the primary source of Al, are included in the list of critical raw materials for the European Union published by the European Commission^26^ in 2020 and because both metals fulfill important functions for EV performance. Co is a crucial element in the cathode of the lithium-ion battery (LIB), which is currently the most widely employed battery type in EVs.^27^ Al plays a significant role as a lightweight material in the structural part of the EV,^28^ is an important wiring material,^29^ and is used in cathode current collectors of LIB cells.^30^ Furthermore, it should be considered that Co and Al supply chains allow for testing SPOTTER by examples of two different types of materials, i.e., an abundant material (i.e., Al) and a scarce material (i.e., Co).

This article is structured as follows: Section 2 provides first an overview of the main elements of the SPOTTER approach and explains then the goal and scope definition, the quantification of inventory flows, the assessment of related impacts, and the interpretation of results in terms of the present case study. In section 3, the results of the case study are presented and discussed, and suggestions are made to mitigate the indicated supply risks. Section 3 concludes by highlighting limitations and future research directions.

## 2 Methods and Materials

### 2.1 Overview of Main Elements of the SPOTTER Approach

SPOTTER^25^ is the first approach that is integrated into the LCSA framework and provides a quantitative assessment of supply disruption impacts along the full supply chain in the short term (i.e., < 5 years) and medium term (i.e., 5 to 15 years). The goal of this approach is to identify supply disruption hotspots, i.e., the biggest supply bottlenecks, and overall supply disruption impacts, i.e., aggregated impacts along the supply chain. To achieve this goal, inventory analysis, impact assessment, and interpretation of the results are performed in analogy to an LCSA. In the stage of inventory analysis, country-specific unit processes within the product system, i.e., processes along the supply chain that occur in different countries worldwide, are defined, and inventory flows that describe the inputs and outputs of these unit processes are collected. In the stage of impact assessment, impacts are evaluated individually for each of the collected inventory flows by multiplying inventory flow amounts with characterization factors (CFs) that define specific supply disruption impacts. The sum of all calculated impact scores is then interpreted as the overall impact, and the highest impact scores are interpreted as hotspots.

The elements considered for the impact assessment within SPOTTER comprise (i) supply disruption events, i.e., changes of conditions affecting the product system, (ii) case-specific CFs representing cause-effect chains between considered supply disruption events and impacts, and (iii) the specific impact categories comprising these impacts. In their method description, Berr et al.^25^ have described the events relevant for a short-term or a medium-term assessment as well as the different indicators required for the calculation of the CFs for defined, pertinent impact categories. In addition, Berr and colleagues^25^ proposed a practical procedure for the application of the SPOTTER approach, the so-called “SPOTTER implementation procedure”. In the Article, this procedure, comprising five steps, is used for the performance of the case study (as shown in sections 2.2 to 2.5).

### 2.2 Goal and Scope Definition

An assessment of short-term impacts along the Co and Al supply chains of EVs used in Switzerland is performed following the “SPOTTER implementation procedure”. The two objectives are (i) identifying relevant supply risks and (ii) calculating scores for the overall supply disruption impact. The functional unit is the Swiss EV fleet in 2019.

Based on the first step of the “SPOTTER implementation procedure”, the focus is set on the causes (i.e., events) and impacts of supply disruptions that can be quantified with the indicators used in SPOTTER (see list of indicators in Berr et al.^25^). The country-specific events “geopolitical instability”, “child labor restrictions”, “trade barriers”, and “depletion of economic resource” as well as the global events “price volatility” and “limited recyclability” are thus considered. Country-specific events refer to changes in conditions affecting the product system that occur in a specific country, while global events represent these changes related to the global market of a material/product. Considered impacts belong to two impact categories, “cost variability”, which refers to the effects of price hikes, and “limited availability”, which represents the effects of physical unavailability. A more in-depth description of the events and impact categories is provided in Berr et al.^25^

**Figure 1:**
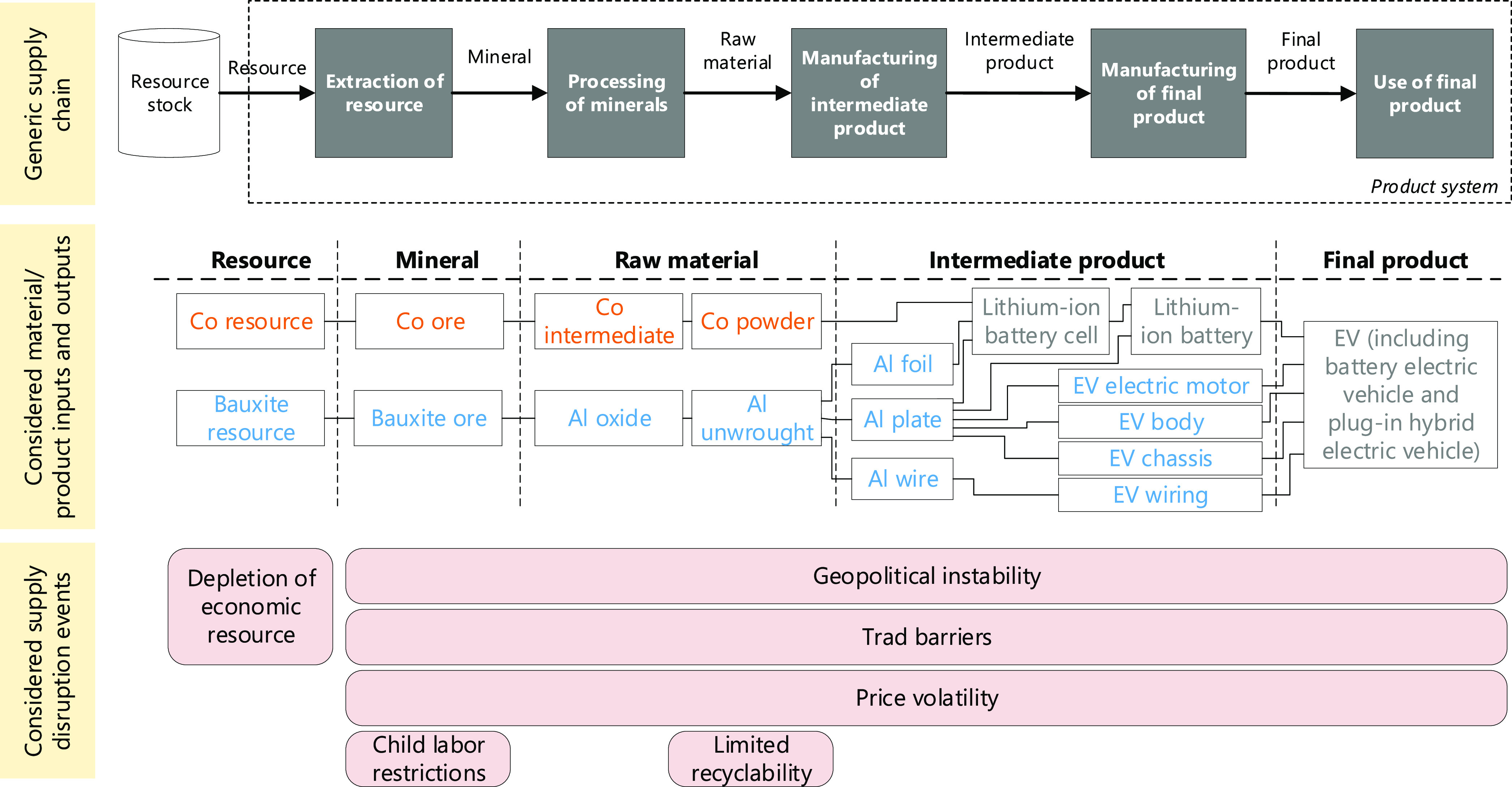
Description of inputs/outputs and supply disruption events considered along the cobalt (Co) supply chain (in orange), the aluminum (Al) supply chain (in blue), or both supply chains (in gray) of electric vehicles (EVs).

The model of the product system comprises supply chain processes related to the extraction of resources, processing of minerals, and manufacturing of intermediate/final products (see upper part of Figure 1). The material- and product-specific inputs and outputs of these processes are represented in the middle part of Figure 1. The choices related to this bill of materials are explained in section S1 of the Supporting Information (SI). The bottom part of Figure 1 illustrates the events that are analyzed at the different supply chain stages. Geopolitical instability, trade barriers and price volatility may lead to disruption of flows along the full supply chain. Conversely, child labor potentially occurs during artisanal mining of Co and bauxite, as reported by Banza Lubaba Nkulu et al.^31^ and Hentschel et al.,^32^ but does probably not take place downstream of the supply chain for high-tech products such as EVs and traction batteries.

The impact assessment with SPOTTER is performed as part of an LCSA framework and, thus, complements environmental impact assessments conducted with traditional process-based life cycle assessment (LCA) studies. In the inventory analysis, inventory flows are quantified by collecting inputs/outputs of materials/products for each supply chain process in relation to the relevant countries and time frame (i.e., information not older than 5 years).

Two different options of data sources have been evaluated in terms of collecting the required information for the inventory analysis (details regarding this evaluation are summarized in section S2 of the SI). As the first option, ecoinvent^33^ is considered, a unit process life cycle inventory database commonly used in LCA studies. Following our evaluation, data from ecoinvent is not sufficient for the inventory analysis because information about specific processes, materials/products, and countries along supply chains is missing and/or is outdated. As a second option, complementing these data with trade data is investigated, based on the suggestion of Beylot et al.,^34^ who assess the environmental impacts of European trade in a process-based LCA study. BACI^35^ is therefore consulted, a database reporting country-specific material/product-level trade data in the form of physical amounts (in kg) and monetary values (in $) for material/product categories described with six-digit harmonized system (HS) codes provided by the World Customs Organization.^36^ BACI is seen as a particularly interesting option because it covers various trade flows along global supply chains, and its data has already been used in several studies for quantifying supply chains, including for example the ones of Sun et al.,^37^ Helbig et al.,^38^ Godoy León et al.,^39^ and Liu and Muller.^40^ Furthermore, the BACI database has also been used in, for example, recent LCSA studies such as the one performed by Siddhantakar et al.,^41^ which is based on the GeoPolRisk approach.^21^ Following our evaluation, the physical trade amounts included in BACI provide sufficient information for inventory analysis. The HS codes relevant for quantifying the considered supply chains are described in section S3 of the SI.

However, an issue with BACI data is the aggregation levels of material and product categories described with relevant HS codes. This aggregation issue is addressed in the present study by using global average market shares and cost-to-mass ratios, since, as shown in section S4 of the SI, the use of such shares and ratios allows for estimating trade flows of specific materials/products. Adjusting the content of the HS codes, however, also adds uncertainty to the results of the study.

### 2.3 Inventory Analysis

The inventory analysis corresponds to the second step of the “SPOTTER implementation procedure”. Figure S1a displayed in section S5 of the SI illustrates the identified unit processes and their inventory flows exemplarily for one part of the supply chain. The inventory flows of each of the identified unit processes are quantified by following the procedure described in Figure S1b. First, the reference amount for the final products is defined, and then weight ratios, trade amounts, and domestic production amounts of the individual materials/products are determined upstream of the supply chain.

Data from BACI is used to define the trade amounts of raw materials and intermediate/final products, and data from the United States Geological Survey^42,43^ (USGS) is utilized to quantify the trade flows of minerals. Additional data sources are consulted to gather data about weight ratios and domestic production amounts. The specific quantification procedure involving also the third and fourth step of the “SPOTTER implementation procedure” (i.e., screening of inventory flow relevance and temporal relevance) as well as the required types of data sources is explained in section S5 of the SI.

The completely quantified supply chain as well as the specific data types and sources used for the quantification are described in the Excel sheet “Inventory flows Swiss EV” provided in the SI.

### 2.4 Impact Assessment

The impact assessment is performed in accordance with the fifth step of the “SPOTTER implementation procedure”, where overall impact scores for the product system (PS) and bottleneck scores are defined. These overall impact scores are calculated by the sum of all bottleneck scores for the individual inventory flows (i.e., Bottleneck score_mat_UP_) as shown in equation 1.
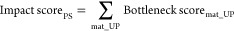
1The bottleneck scores refer to supply bottlenecks along the supply chain. These scores are calculated by multiplying the inventory flow amount (*m*_mat_UP_) with respective CFs (CF_mat_UP_) as shown in equation 2. As shown by Berr et al.,^25^ these CFs describe cause–effect chains between the six events and two impacts of supply disruptions listed in section 2.2. They are calculated based on the following four basic indicators: (i) indicator for supply disruption event over a period (EI*t), (ii) indicator for supply diversity (DI), (iii) indicator for vulnerability to physical shortage (PVI), and (iv) indicator for economic importance or damage (EVI). EI*t and DI are summarized in an indicator for the supply disruption probability over a period (PI*t). The values of the PIs are then consistently scaled based on a min–max-scaling described by Berr et al.^25^ to allow for an aggregation of bottleneck scores calculated for the individual events into the two impact categories.
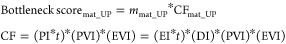
2

As explained by Berr et al.,^25^ the PI and EVI values are context dependent and, thus, case-specific CFs are calculated. The calculation of the CFs and bottleneck scores used in the present study is explained in section S6 of the SI. A Python script that has been developed within our work and the open source software Brightway2^44^ have been used for the required calculations.

### 2.5 Interpretation

Two kinds of hotspots, i.e., hotspots per impact category (e.g., hotspots of cost variability) and hotspots per individual supply disruption event and impact category (e.g., hotspots of cost variability due to geopolitical instability), are defined. The first kind of hot spot is defined by considering all bottleneck scores that are higher than 1% of the overall impact per impact category. The second kind of hotspot is defined by considering all bottleneck scores that are higher than 1% of the overall impact per supply disruption event and impact category. The threshold of 1% is set following the “Guide for Interpreting Life Cycle Assessment Results” published by Schau et al.,^45^ where contributions above 1% of the total impact are highlighted as relatively high.

## 3 Results and Discussion

### 3.1 Locations of Supply Disruption Hotspots Per Impact Category

**Figure 2:**
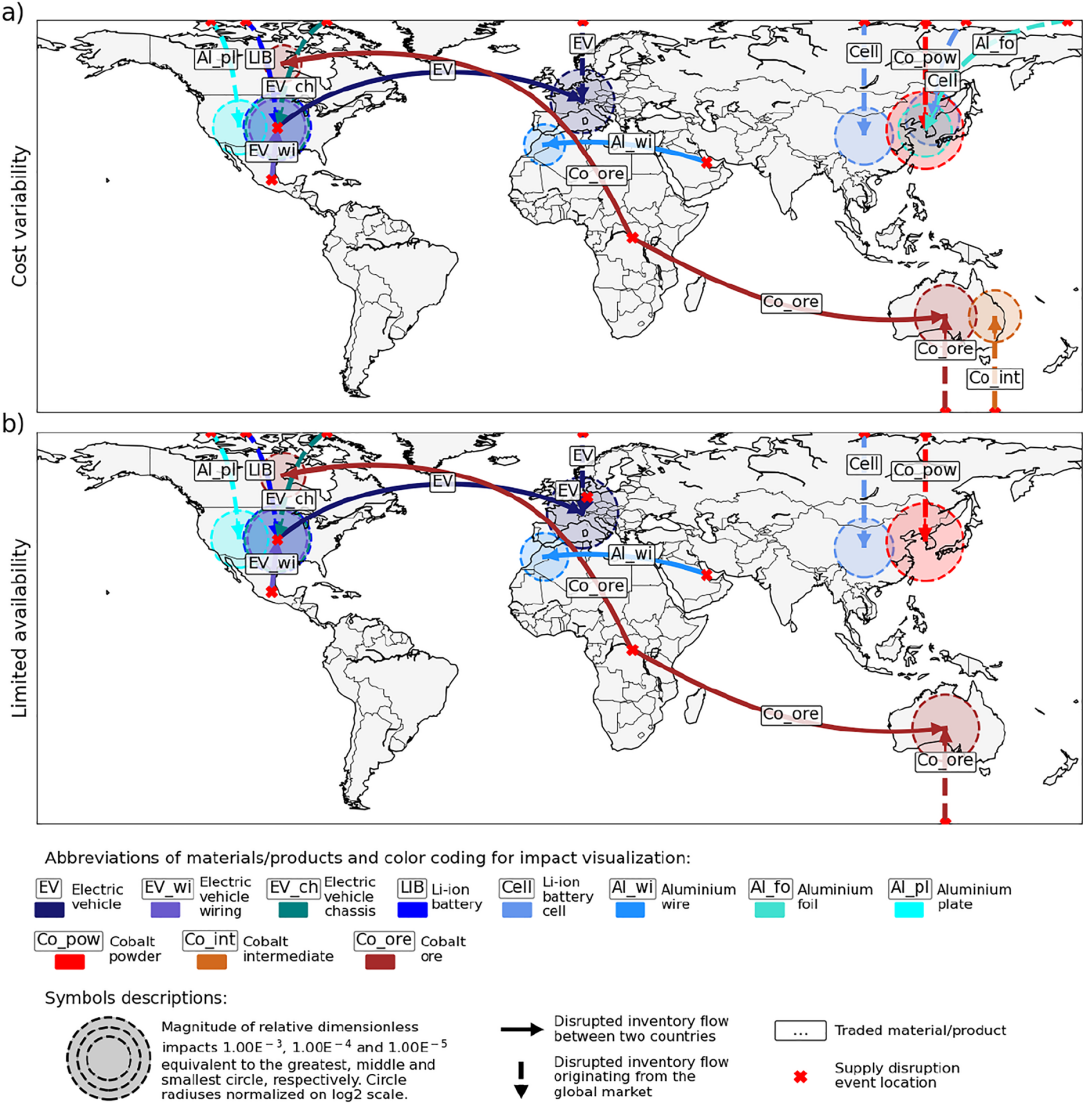
Hotspots of (a) cost variability and (b) limited availability along the cobalt and aluminum supply chain of electric vehicles used in Switzerland. Hotspots are visualized with red/brown color shades for upstream stages of cobalt supply and with blue/purple color shades for downstream supply chain stages.

Figure 2 and Figure 4 use global maps as a presentation format to illustrate geographical locations of the identified supply disruption hotspots. The two maps shown in Figure 2 display impacts higher than 1% of the overall impact scores for cost variability and limited availability (i.e., impacts referred to as the first kind of hotspots in section 2.5).

Within the global maps, locations of hotspots due to country-specific supply disruption events are indicated with solid arrows that range from countries in which the event occurs to the countries affected. Conversely, locations of hotspots due to global events are marked with vertical dashed arrows that reach from the top or the bottom of the map to the affected countries. The magnitude of the impacts is described by the size of circles placed on top of the affected country. Locations of events are indicated with red crosses.

Three-quarters of the hotspots presented in Figure 2 (i.e., 12 out of 16) are hotspots of both cost variability and limited availability. Two examples are impacts of EVs traded from the USA to Switzerland and impacts of LIB cells supplied from the global market to China. This suggests that there is a correlation between impacts covered by the two categories. Such a correlation has also been identified by Frenzel et al.,^46^ who state that particularly large effect sizes of price hikes lead to severe physical disruptions. Information on effect sizes specific to price hikes and physical disruptions would thus allow for assigning impacts to cost variability or limited availability, but as also stated by Frenzel et al.,^46^ such information is still widely missing. Another explanation for the correlation between the impacts is related to calculations of impact scores. Calculations of scores for the two impact categories differ in only one of four indicators, i.e., the indicator for economic importance or damage. Hence, when the values of the other three indicators are pivotal for the impact assessment, the calculated bottleneck scores inevitably refer to impacts of both categories.

The remaining quarter of the hotspots is specific to cost variability or limited availability. Hotspots related to cost variability indicate flows of materials and products with specifically high economic importance for the product system. The flow of LIB cells from the global market to Korea is an example of such a flow. Conversely, hotspots related to limited availability suggest relatively large affected revenues. The revenue related to EVs traded from Germany to Switzerland is an example of such an affected revenue.

Several hotspots (i.e., 12 out of 16) refer to the supply of intermediate/final products. These hotspots often indicate supply risks along the supply chains of one specific end-product manufacturer. For example, potential disruptions of EV wiring supply from Mexico to the USA affect only the supply chains of US EV manufacturers. In this case, the restructuring of the supply chain by importing EVs also from countries other than the USA may be a viable risk mitigation measure. In the case of supply risks indicated with the remaining hotspots, supply chain restructuring may not be useful because the described potential disruptions of raw materials and minerals supply often affect simultaneously the supply chains of several end-product manufacturers. For example, potential disruptions of Co ore supply from Congo to Australia supposedly affect the supply chains of EVs produced in Germany and the USA. Measures suitable for dealing with these and other supply risks identified with our hotspot analysis are suggested in section 3.4.

### 3.2 Relative Magnitude of Hotspots

**Figure 3:**
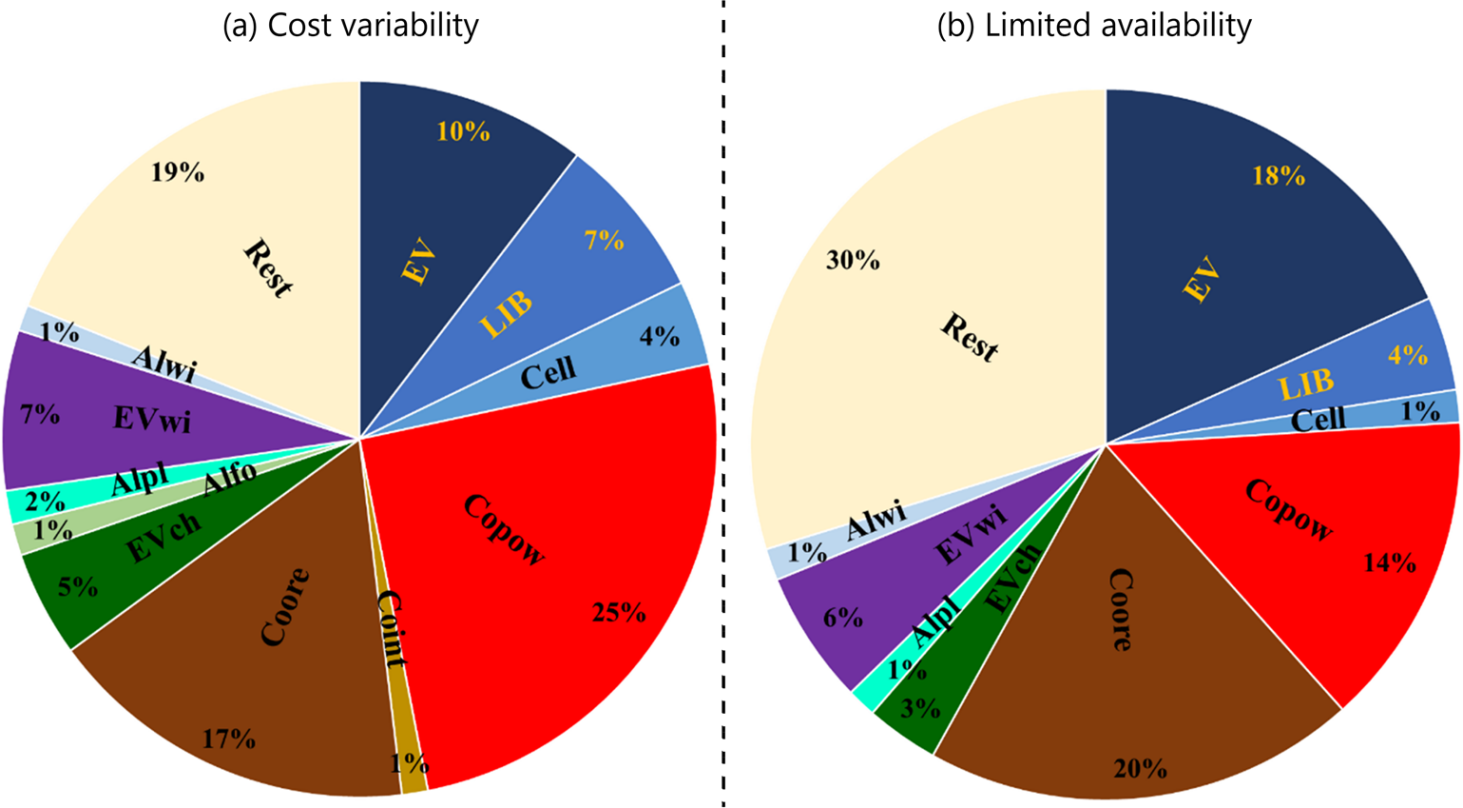
Magnitude of hotspots for electric vehicles used in Switzerland considering (a) the cost variability and (b) the limited availability for materials/products used along the cobalt and aluminum supply chains. Abbreviations for the materials and products are explained in Figure 2.

While the maps shown in Figure 2 are useful to represent the locations of hotspots, they do not allow for clearly illustrating the relative magnitude of specific hotspot scores and thus make it difficult for decision-makers to identify the most relevant hotspots. The pie charts shown in Figure 3 present the shares of the hotspot scores for cost variability and limited availability aggregated on the level of the affected material/products. Impacts that are not classified as hotspots (i.e., represent less than 1% of the overall impact) are summed up in the category “Rest” (beige color). The hotspot shares related to individual flows of the materials/products are visualized in section S7 of the SI, where these shares are presented for hotspots per impact category and hotspots per individual supply disruption event and impact category in stacked bar charts.

Overall, the highest contributions to the overall impacts are associated with hotspots related to the supply of EVs, EV wiring, traction batteries and their cells, and Co powder as well as Co ore. The shortage of wiring and traction batteries for car manufacturers, the potential cost increases of Co powder, as well as the insecurity related to the supply of Co ore from Congo have been highlighted in various media.^47−50^ Identified hotspots indicated with the highest contributions in Figure 3 are thus in line with current or predicted future concerns of supply chain managers. The particularly high contributions of EV impact to the overall impacts (around 10% in the case of cost variability and around 18% in the case of limited availability) may however seem odd since EV shortage is not considered a big issue in the real world. An explanation for this difference in perception is that our study considers EVs as the only available vehicle type and disregards the purchase of conventional vehicles. As more conventional vehicles than EVs are currently on the market and in use in Switzerland, considering conventional vehicles as an alternative to EVs would certainly lower the physical availability constraints and thus the impact of EVs. However, following the Clean Vehicles Directive implemented by the European Parliament and Council^51^ regarding the phasing out of petrol and diesel cars by 2035, purchasing conventional vehicles does not really describe a reasonable alternative in the future.

### 3.3 Locations of Supply Disruption Hotspots per Event and Impact Category

**Figure 4:**
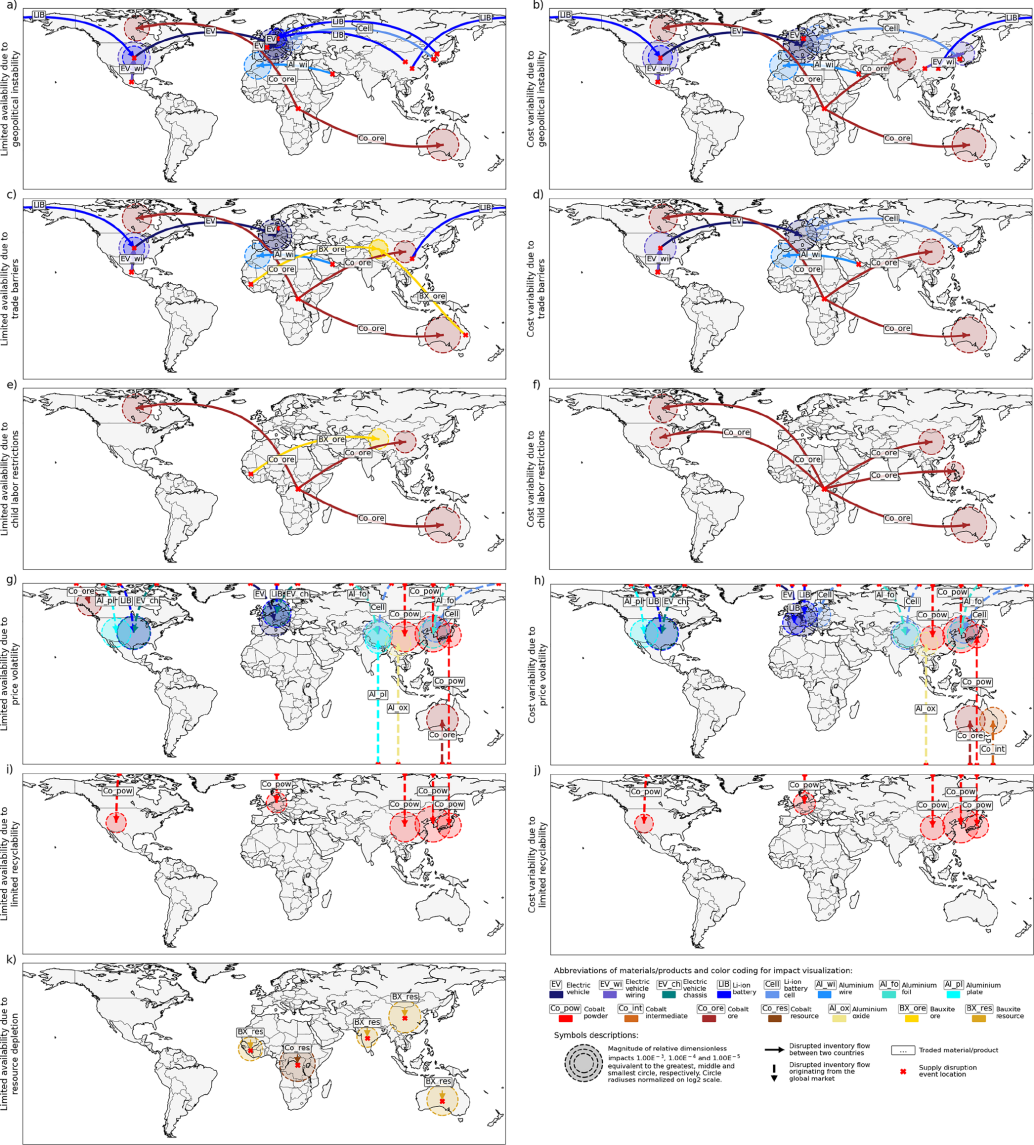
Hotspots of limited availability (left) and cost variability (right) caused by individual events considering six different supply disruption events along the cobalt and aluminum supply chain of electric vehicles used in Switzerland. Hotspots are visualized with red/brown color shades for upstream stages of cobalt supply, with yellow color shades for upstream stages of aluminum supply and with blue/purple color shades for downstream supply chain stages.

The 11 maps shown in Figure 4 display a disaggregated version of Figure 2, which represents impacts specifically for the individual events (i.e., impacts referred to as second kind of hotspots in section 2.5). The related impact scores are higher than 1% of the scores for cost variability or limited availability caused by individual supply disruption events.

Figure 4a–f and k suggest that supply chains may often be disrupted due to events originating in Asian, African, or other developing countries and affecting Western economies. The identified hotspots are, for example, related to material/product flows from China, Korea, Mexico, Guinea, and Congo to the USA, Canada, Germany, and Poland. Reasons for these hotspots are the high probability of occurrence of supply disruption events in developing countries,^10,52,53^ the concentrated trade of materials/products in these countries,^54^ and/or the high dependency of Western economies on the supply of these materials/products.^55^

As shown in Figure 4a–f, some inventory flows may be disrupted due to the occurrence of multiple events. In some cases, the likelihood of the occurrence of different events in the same country is particularly high, as seen, for example, by the events causing potential disruptions of Co ore supply from Congo to Australia. The World Bank^10,52^ and Benoit Norris et al.^53^ rate the probability of geopolitical instability, trade barriers, and child labor restrictions for Congo as relatively high. In other cases, the supply concentration or vulnerability factors have a high influence on the impact, as seen in the example of risks related to EV wiring supply from Mexico to the USA. The probability that supply disruption events occur in Mexico is rated as relatively low by the World Bank,^10,52^ but the influence of market concentration and economic importance or damage is relatively high in this example.

The previous example highlights that some impacts constitute hotspots, because related supply disruption events have relatively large consequences but rather low probabilities of occurrence. The identified hotspots related to the EV supply from Germany to Switzerland are such an example, as the occurrence of geopolitical instability and trade barriers is seen as rather unlikely for Germany, but the German EV imports are considered to be of high economic importance. Furthermore, the hotspots related to the Al wire supply are another example of hotspots, which might be surprising as they indicate supply disruption risks that, in times of several extensive disruptions along the EV supply chains (see examples in section 1), do not manifest in the real world. The reasons why they have been identified as hotspots are a relatively high probability of geopolitical instability and trade barriers in Bahrain, the Al wire market concentration on the flow from Bahrain to Morocco, as well as the high economic importance/damage related to the disruption of this flow.

The majority of hotspots due to geopolitical instability and trade barriers are defined by impacts of intermediate and final products (Figure 4a–d). One reason is related to the fact that only impacts of traded materials/products are evaluated for these two events, while domestic production is considered “risk-free”. Indeed, the impacts of Co powder and unwrought Al are low because both metals are mainly domestically refined.^56,57^ Conversely, intermediate/final products such as LIBs, EV wiring, and EVs are frequently traded because specialized production processes are often spread over different countries. Their production has become increasingly specialized to enhance productivity, competition, and innovation.^58^ In relation to such specialization, the supply of intermediate and final products is often concentrated in a few countries. An example is the supply of LIBs, of which 72% are produced in China according to Yu and Sumangil.^59^ The supply of Co powder and unwrought Al in turn is relatively diverse following the BACI trade data, i.e., exports are distributed over different countries. As these two raw materials have thus a relatively low market concentration, their impacts are considered comparably low. The remaining hotspots due to geopolitical instability and trade barriers refer mainly to the supply of Co ores from Congo, as stated above, a particularly unreliable source, but also to flows of bauxite ores from Guinea and Australia to China.

Hotspots due to child labor restrictions (Figure 4e and f) are, as explained in section 2.2, analyzed only for the Co and bauxite ore supply in our study. These hotspots are mainly related to the Co ore supply from Congo but also occur for the bauxite supply from Guinea. Benoit Norris et al.^53^ estimate very high risks of child labor for both Co and bauxite mining. The reason for the dominance of hotspots related to Co supply in Figure 4e and f is the higher country concentration of Co mining in relation to bauxite mining indicated by the USGS.^60,61^ Banza Lubaba Nkulu et al.^31^ report the potential occurrence of child labor during the widespread artisanal mining of Co ore in Congo, and the U.S. Department of Labor, Bureau of International Labor Affairs^62^ documents child labor in bauxite mining in its report, which aligns with the results of our hotspot analysis.

Hotspots due to price volatility (Figure 4g and h) are defined by impacts of materials/products with relatively high price variations and particularly large trade or domestic production amounts. Here, mainly Asian, European, and Northern American countries are affected as large amounts of materials/products are consumed for the production and manufacturing processes in these countries. Following Figure 4g and h, price volatilities are mostly associated with the materials/components of traction batteries such as battery cells, Al foil, and Co powder. An example of a hotspot is the LIB cells used for LIB production in China, for which relatively volatile prices are seen following BACI trade data and are reported by BloombergNEF.^63^

Hotspots due to limited recyclability of raw materials (see Figure 4i and j) are only defined by the impacts of Co powder. Co powder has a lower recycling rate than unwrought Al according to Church and Wuennenberg^64^ and The Aluminum Association.^65^ By following the min-max-scaling described by Berr et al.,^25^ related supply disruption probabilities are rated with 100% for Co powder and 0% for unwrought Al. All impacts caused by limited recyclability of unwrought Al are thus evaluated as zero. The identified hotspots are concentrated in Asia, as Co powder is mainly used in Asia for the production of traction batteries.

Hotspots due to resource depletion (see Figure 4k) are located in countries where the extraction-to-resource stock ratios and/or the extracted resource amounts are relatively high. Co resources extracted in Congo and bauxite resources extracted in China and Australia thus describe the major hotspots. However, while the use of the here-applied indicator is suggested by Berger et al.^66^ to assess impacts of resource depletion on the product system, the related hotspots should generally be treated with caution, as resource depletion within the next five years is rather unlikely. Jowitt et al.^67^ for example have highlighted that global resource stocks of Co and bauxite have not significantly decreased in relation to production over the last 50 years. The intention behind presenting these hotspots is thus not to inform about the unavailability of resources but to highlight the locations in the supply chain where price increases for resource extraction processes would have the highest impacts on the product system. This issue could probably be described more appropriately with other indicators than the extraction-to-resource stock ratios, but, as the review of Sonderegger et al.^68^ shows, such indicators are currently not available in the literature.

### 3.4 Possible Risk Mitigation Measures

Last but not least, possible measures for mitigating the supply risks identified with the support of our hotspot analysis (see preceding sections) are listed here. As short-term impacts along Swiss supply chains have been assessed in our study, the proposed measures are targeted toward the designers of the Swiss resource strategy for the next 5 years as well as toward Swiss retailers of EVs. The suggested mitigation measures have been identified by making use of the list of generic risk mitigation measures presented in the report of Spörri et al.^69^

The identified supply risks are split into three different groups. The first group of identified supply risks refers to potential disruptions of the EV supply. These disruptions could result from price volatilities, geopolitical instabilities, and trade barriers in the USA and Mexico affecting the supply of EVs and EV components. As mentioned in section 3.2, supply disruptions of EVs have so far not been a big concern, as conventional vehicles could be purchased instead of EVs. However, when EVs are increasingly implemented as replacements for conventional vehicles as predicted by, for example, BloombergNEF,^70^ it becomes crucial to establish risk mitigation measures. Risks related to the price volatilities of EVs could be addressed by implementing hedging strategies, and the dependency on EVs produced in the USA could be reduced by restructuring supply chains as suggested in section 3.1.

The second group of identified supply risks refers to supply disruptions of traction batteries or battery cells supplied by Asian countries, as shown in Figure 4a–d. In the case of these supply risks, restructuring the supply chain may not be a viable option for risk mitigation, as these batteries are already integrated into the vehicle by EV manufacturers in various countries. Instead, policy makers could incentivize circular economy strategies for Switzerland regarding traction battery supply by supporting related research activities and the establishment of required infrastructure as already done for example in the frame of the CircuBAT project.^71^ Due to the nonexistent EV production in Switzerland and the resulting complete reliance on EV imports from abroad, Swiss industry stakeholders are limited in their possibility to establish mitigation measures. However, EV producers in other countries could conclude long-term contracts with traction battery suppliers that are located in trustworthy countries such as most of the European countries (see list of national reputation ratings published by Knoema^72^) or establish backward integration for their battery supply. As mentioned before, Swiss retailers could then restructure their supply chain by increasingly buying from more reliable EV producers.

The third group of identified supply risks refers to potential supply disruptions for EV materials/components caused by price volatilities, limited recyclability, and country-specific events (i.e., geopolitical instability, trade barriers, or child labor restrictions). Following Figure 2 and Figure 4, such disruptions are particularly likely along the supply chain of Co. To mitigate these risks, policy makers could support research activities on (further) developments of the chemistry of the traction batteries aiming, among other things, for a reduction of the battery’s cobalt content. First research activities in this direction are already performed, for example, within the “SeNSE” project.^73^ Furthermore, research on more effective recycling of critical materials such as Co from traction batteries, activities that have already been initiated according to the Federal Laboratory for Materials Testing and Research.^74^ Furthermore, battery and EV producers could build up stockpiles of the most critical materials and components needed for their production process, which constitutes a measure that has also been suggested by Sprecher et al.^14^ to tackle supply risks of critical metals in the short term.

As shown above, our hotspot analysis allows for highlighting potential supply disruptions along the full supply chain. Following the comparison between the results of our study and the ones of existing studies described in section S8 of the SI, existing studies, in contrast to our study, only consider parts of the supply chain and do not inform about country-specific variabilities of impacts. Hence, some of the recommendations for risk mitigation provided in this section could not have been deduced from existing studies. For example, an effective restructuring of supply chains or the conclusion of long-term contracts with producers can only be carried out when the most critical material/product flows between the different countries along the supply chain are known.

### 3.5 Limitations and Future Research

In this article, we have demonstrated the application of the SPOTTER approach for the assessment of short-term impacts of supply disruptions along the cobalt and aluminum supply chains of electric vehicles (EVs) used in Switzerland. We then discussed the results of this more comprehensive analysis of supply disruption hotspots along the supply chain (compared to existing studies) and finally illustrated how these results could facilitate the identification of suitable risk mitigation measures.

Nevertheless, certain limitations remain that need to be addressed in future research. First, there are issues concerning the quantification of event probabilities, as the currently used indicators may not adequately represent the supply disruption event (see for example the discussion regarding resource depletion indicators in section 3.3) or the provided scales of indicators may lead to an over- or underestimation of probabilities. To tackle these issues, the use and definition of related indicators could be refined. With regard to, for example, the resource depletion indicators, empirical studies in collaboration with mining companies could be performed to acquire pertinent data regarding economic resource stocks. Second, the quality of the assessment results is highly sensitive to the data availability and quality. This issue could be addressed by extending databases used in criticality assessment and life cycle sustainability assessment with more detailed material/product flow information acquired from, for example, relevant scientific articles or reports. Third, the relative importance of identified supply disruption hotspots has been determined based on the aggregation of bottleneck scores into overall impact scores. While a linear relationship between these bottleneck scores is assumed, which does not necessarily exist, another possibility to analyze the relative importance of hotspots would be to cross-check the results with industry experts as suggested by Schrijvers et al.^75^

In the study presented here, we have applied SPOTTER for a hotspot analysis on the product level. Future research could focus on performing further types of assessments with SPOTTER. One future research direction could be the identification of supply scenarios associated with comparably low supply risks by comparing the overall impact scores related to each scenario. Scenarios could, for example, be designed considering changes in the Swiss EV fleet or the supply situations for EVs used in other countries. Another future research direction could be to assess the impacts related to specific flows along the supply chain before and after their disruption. This would allow for an evaluation of whether the supply chain has become more resilient through response to supply disruptions.

While the focus of the presented case study has been on identifying supply disruption impacts of the cobalt and aluminum supply chains of EVs, in the next step, the application of SPOTTER will be extended toward an assessment on a sectoral level. The objective of such an assessment will be to analyze hotspots along the supply chains of all of the critical raw materials used within technologies relevant to different sectors and to compare impacts between different technologies.

## Data Availability

All underlying data that are used to generate the results in this paper are available in the main document or the PDF and Excel files attached as Supporting Information. The original code is not reported in this paper, but it can be made available for academic purposes from the lead contact upon request. A license for the Social Hotspot Database (http://www.socialhotspot.org/) is necessary to provide the data related to indicators for child labor restrictions. All other data required to regenerate the results in this paper are openly accessible and can be requested from the lead contact.

## Supporting Information Available

Explanations of the considered bill of materials/products that is considered for the supply chain model; evaluation of data sources suitable for the quantification of the considered supply chains; identification of the harmonized system codes that are suitable to represent the considered bill of materials; explanation of adjustments related to the content of the considered harmonized system codes; explanation of the procedure that is applied for the quantification of the supply chain; demonstration for the calculation of considered impact scores and characterization factors based on specific examples; visualizations of the relative magnitude of hotspots related to individual supply disruption events (PDF)
Calculations and data to define the inventory flows (XLSX)
Depletion potential indicator (XLSX)
Geopolitical instability indicator (XLSX)
Price volatility indicator (XLSX)
Trading across borders indicator score (XLSX)
LCIA data LIB and EV (XLSX)
LCIA data EV body, motor, chassis and wiring (XLSX)
LCIA data Al foil, plate and wire (XLSX)
LCIA data Co mining and processing (XLSX)
LCIA data Al ore, oxide and unwrought (XLSX)
