# Supplementary material for: Assessing Short-Term Supply Disruption Impacts within Life Cycle Sustainability Assessment—A Case Study of Electric Vehicles
Source: Environ Sci Technol. 2023 Nov 13;57(48):19678–89. doi: 10.1021/acs.est.3c05957 (PMC10702435; doi:10.1021/acs.est.3c05957)
Supplement: Supplementary file 1 — es3c05957_si_001.pdf [file es3c05957_si_001.pdf]

Supporting information

Assessing Short-Term Supply Disruption Impacts within Life Cycle Sustainability Assessment – a Case Study of Electric Vehicles

Marcus Berr[a], Roland Hischier[a], Patrick Wäger[a]*

[a]Empa, Swiss Federal Laboratories for Materials Science and Technology, Lerchenfeldstrasse 5, 9014 St. Gallen, Switzerland

**Corresponding author. E-mail address:* marcus.berr@empa.ch (M. Berr).

Number of pages: 33

Number of figures: 4

Number of tables: 3

Content

S1. Explanation of the considered bill of materials/products ... 2
S2. Identification of suitable data sources ... 4
S3. Relevant Harmonized System Codes ... 6
S4. Adjustments related to the content of considered Harmonized System Codes ... 7
S5. Procedure for the quantification of the supply chain ... 11
S6. Calculation of bottleneck scores and characterization factors ... 16
S7. Relative magnitude of hotspots for each impact type ... 22
S8. Comparison with existing studies ... 28
References: ... 30

S1. Explanation of the considered bill of materials/products

As shown in Figure 1 of the main article, different material/product inputs/outputs along the cobalt (Co) and aluminium (Al) supply chain of electric vehicles (EVs) are considered to describe the product system. The choices regarding the bill of materials are explained in this section.

Following Irle[1], the considered EV types comprise battery electric vehicles (BEVs) and plug-in hybrid electric vehicles (PHEVs). These two types of EVs have been selected because they offer the potential for carbon-free transport operations, which are important in terms of the decarbonization of the mobility sector.[2, 3]

BEVs and PHEVs are typically equipped with a lithium-ion battery (LIB) that consists of a battery case and multiple battery cells. Each of these cells includes a cathode, an anode, a current collector, a circuit and an electrolyte.[4] Other components of the EVs are bodies, chassis, electric motors and electrical systems consisting of stranded wires.[5, 6] According to PrimecomTech[7], AC or DC motors can be used for EVs. Following Schröder[8] and Matt et al.[9], DC electric motors with a power output between 750W and 375kW and AC electric motors with a power output between 10 kW and 100 kW are suitable for being used in EVs.

LIBs, bodies, chassis, electric motors and wiring of EVs are considered in the presented case study because they utilize Co and Al. The use of Co and Al in these EV components is explained in the next paragraph. Other EV components such as the internal combustion engine of PHEVs and the paint of EVs are neglected for the case study because Co and Al are usually not used for their production. Al alloys are typically not used in the internal combustion engines of the PHEVs, because, according to Sullivan et al.[10], iron and steel are the preferred materials in these engines due to better noise suppression. While Al powder is sometimes used in the painting of the car, it is, according to International Driving Authority[11], not a crucial constituent of the painting.

As mentioned before, the raw materials of Co and/or Al are part of the LIBs, bodies, chassis, electric motors and wiring of EVs. Cobalt powders and Al foils are commonly used within the cathode of the LIB cells.[4, 12] Alloyed Al plates are typically utilized in the frames of the LIBs, bodies, chassis and electric motors of EVs.[13-17] Al alloys are usually deployed in wires for the electrical system of the EVs.[18]

As shown by Baars et al.[19], the pure Co powder used in the LIBs of the EVs is produced through the extraction of Co resources from the resource stock as well as through processing of Co ores and Co intermediates. Following Schmidt et al.[20], Co powder can be produced from three different types of Co intermediates, i.e. from Co mattes, from nickel-cobalt mattes or from nickel-cobalt sulfides. As illustrated by Liu and Muller[21] and Liu et al.[22], the production of unwrought Al used in the different EV components involves the extraction of bauxite resources as well as the processing of bauxite ores and Al oxides.

S2. Identification of suitable data sources

In this section, two different databases are evaluated regarding their suitability to quantify the considered Co and Al supply chains. The first database is ecoinvent, the globally most detailed unit process life cycle inventory database.[23] The second database is BACI, a database reporting physical and monetary country-specific trade data for different material/product categories.[24]

The two databases need to fulfill the following four criteria in order to be suitable for quantifying the considered supply chains:

i. availability of information for the technology type of relevant processes along the supply chain
ii. availability of information for inputs/outputs of the relevant materials/products
iii. availability of information for the relevant countries or geographical regions
iv. availability of up-to-date information

ecoinvent provides information for some specific processes along the supply chain (e.g. "cobalt production" with an output of Co powder), but information for other processes such as "processing of Co ore" or "production of BEVs" is missing. Material/product inputs and outputs are stated for each process that is included in ecoinvent. However, due to the lack of certain processes in the database, some of the relevant inputs/outputs are missing. Furthermore, geographical descriptions are often only available on a global level or for large regions (e.g. rest of the world) and thus, ecoinvent lacks sufficient country-specific information. Finally, some of the datasets are outdated (i.e. covering information that is older than 5 years). For example, at the time of our evaluation, a dataset for LIB cell production only exists for the year 2010, which is an issue especially with regard to fast developing technologies such as LIBs. In conclusion, ecoinvent is evaluated as unsuitable to be used for the quantification of the considered supply chain.

BACI covers trade data for more than 5'000 material/product categories related to 6-digit HS codes provided by the World Customs Organization[25]. The database receives annually updated bilateral trade flows for these categories from more than 200 countries. Based on these trade reports, BACI publishes physical trade amounts (measured in kg) and monetary trade values (measured in $). To estimate the volume of trade when only monetary values are reported, a standard unit value is defined for each

material/product category included in BACI. This standard unit value is calculated based on the median unit value of available ratios between monetary values and physical amounts.[26] Comparing the information in this database against the four criteria stated above, the datasets included in BACI that cover physical trade flows are seen as sufficient to be used for the quantification of the considered supply chains. The list of the HS codes that are thus considered for the case study is provided in section S3.

However, an issue concerning the aggregation levels of some of the relevant materials/products along the considered supply chains remains related to the datasets included in BACI. Trade amounts of Co mattes (approx. 27% Co content) and Co powder (approx. 100% Co content), which are actually produced by different processes along the supply chain, are covered within the same HS code, namely 810520 "Cobalt; mattes and other intermediate products of cobalt metallurgy, unwrought cobalt, powders".[27] Furthermore, trade amounts of LIB cells are covered together with cells of lead-acid batteries and nickel metal hydride batteries as well as other parts of electric accumulators such as containers and covers within the HS code 850790 "Electric accumulators; parts n.e.s. in heading no. 8507".[14, 25] Following the approach introduced by the European Commission[28], the trade data related to the individual materials/products within the two HS codes 810520 and 850790 have been identified by using cost-to-mass ratios. The adjustments related to the content of these two and all other considered HS codes are explained in section S4.

S3. Relevant Harmonized System Codes

Table S1 states the identified HS codes for each of the materials/products that have been described in section S1.

Table S1: Considered 6-digit Harmonized System (HS) codes related to the aluminium and cobalt supply chains of electric vehicles. Abbreviations: Cobalt (Co), Aluminium (Al), Lithium-ion battery (LIB), Electric vehicle (EV), Battery elec-tric vehicle (BEV), Plug-in hybrid electric vehicle (PHEV)

Material/Product	HS codes	Description
Co intermediates (Co mattes, Nickel-cobalt mattes, Nickel-cobalt sulfides)	810520	Cobalt; mattes and other intermediate products of cobalt metallurgy, unwrought cobalt, powders
	750110	Nickel; nickel mattes
	750120	Nickel; oxide sinters and other intermediate products of nickel metallurgy
Al oxide	281820	Aluminium oxide; other than artificial corundum
Co powder	810520	Cobalt; mattes and other intermediate products of cobalt metallurgy, unwrought cobalt, powders
Al unwrought	760110 / 760120	Aluminium; unwrought, (not alloyed) / unwrought, alloys
Al foil	760719	Aluminium; foil
LIB cells	850790	Electric accumulators; parts
Al plate	760612 / 760692	Aluminium; plates, sheets and strip
Al wire	760521 / 760529	Aluminium; alloys, wire
LIB	850760	Electric accumulators; lithium-ion
EV body	870710	Vehicles; bodies for electric vehicles
EV chassis	870600	Chassis; fitted with engines, for the motor vehicles
Electric motors	850132 / 850133	Electric motors and generators; DC
	850152 / 850153	Electric motors; AC motors, multiphase
EV wiring	854430	Insulated electric conductors; wiring sets
BEV, PHEV	870380	Vehicles; with only electric motor for propulsion /
	870370 / 870360	Vehicles; with both compression-ignition or spark-ignition internal combustion piston engine and electric motor for propulsion, capable of being charged by plugging to external source of electric power

S4. Adjustments related to the content of considered Harmonized System Codes

Some of the considered 6-digit HS codes cover materials/products that are not only part of the supply chains of EVs but also part of the supply chains of other products. Information provided by the World Customs Organization[25] has thus been used to identify the type of materials and products that are covered within the respective HS codes. If materials/products that are not part of the Co or Al supply chains of EVs are covered within a certain HS code, the content of this HS code has been adjusted. These adjustments have been done based on global average market shares and global average cost-to-mass ratios. To simplify the procedure of adjusting the HS codes content, considered market shares are generally applied to both, the trade amounts and the trade costs of materials/products. It is thus assumed that materials/products covered within the same HS code generally have equal cost-to-mass ratios. If relevant materials/products associated with different cost-to-mass ratios are covered within the same HS code, their respective cost-to-mass ratios are estimated and considered for the adjustment of the HS code content.

The adjustments of HS code contents performed in the frame of the presented case study are described in the following paragraphs.

Battery electric vehicles (BEVs) covered by the HS code 870380 and plug-in hybrid electric vehicles (PHEVs) covered by the HS codes 870370 & 870360:

The considered HS codes cover only products that are relevant for the supply chain of EVs.

Lithium-ion batteries (LIBs) covered by the HS code 850760:

The considered HS code covers besides LIBs that are used in EVs also LIBs that are used in the stationary and the electronics sector. U.S. Department of Energy[29] and Tsiropoulos et al.[30] state average market shares of LIBs used in different end-use sectors. Considering these shares, 60% of the amount and the costs of LIBs covered by the HS code is assumed to be used for the manufacturing of EVs.

LIB cells covered by the HS code 850790:

The considered HS code covers battery modules as well as battery cells that are used in different types of batteries, including cells of LIBs, lead acid batteries (PbAcBs), nickel-metal hydride batteries (NMHBs)

and other batteries. These batteries are applied in the electro mobility, stationary and electronics sectors. A share of 60% of these batteries are included in the electro mobility sector according to U.S. Department of Energy[29] and Tsiropoulos et al.[30]. Grand View Research[31] states market shares of all three battery types in the electro mobility sector (i.e. LIBs: 60%, PbAcBs: 30%, NMHBs: 5%, other batteries: 5%). Cost-to-mass ratios of the different battery cells are used to identify the trade amounts and costs of LIB cells. These ratios are calculated based on the energy densities reported by Wong and Chan[32] (i.e. LIBs: 0.16kWh/kg, PbAcBs: 0.035kWh/kg, NMHB: 0.07kWh/kg) and the cost-to-energy ratios reported by Mongird et al.[33] (i.e. LIB: 271$/kWh, PbAcBs: 260$/kWh, NMHB: 600$/kWh). Following BloombergNEF[34], it is assumed that the prices of battery packs are 70% of the prices of battery cells. The resulting cost-to-mass ratios are 6 $/kg for PbAcB cells and cases, 29 $/kg for NMHB cells and cases and 30 $/kg for LIB cells and cases. Thus, the following three requirements are considered: (i) all trades with a cost-to-mass ratio below or equal to 6$/kg are assumed to be only PbAcB cells and cases, (ii) all trade with a cost-to-mass ratio between 6 and 30$/kg are assumed to be 60% LIB cells and cases, 30% PbAcB cells, 5% NMHB cells and cases and 5% other battery cells and cases and (iii) all trades with a cost-to-mass ratio above or equal to 30$/kg are assumed to be 90% LIB cells and cases and 10% NMHB and other battery cells and cases.

Cobalt powder covered by the HS code 810520:

The considered HS code covers Co powder used in EVs but also in other products and additionally covers Co mattes. As stated in the previous paragraph, 60% of all batteries are estimated to be used in the electro mobility sector. According to Petavratzi et al.[35], 61% of the Co materials that are covered by the HS code 810520 is Co powder used in LIBs. Cost-to-mass ratios of Co powder and mattes are used to identify the trade amounts and costs of Co powder. These ratios are defined based on the average market price of cobalt metal (i.e. 26$/kg on the 29. July 2019 according to London Metal Exchange[36] and Trading Economics[37]) and based on the average market price of Co mattes (i.e. 11$/kg according to Baars et al.[19] and the European Commission[38]). Thus, the following three requirements are considered: (i) all trades with a cost-to-mass ration below or equal to 11$/kg are assumed to be Co mattes, (ii) following Baars et al.[19], all trades with a cost-to-mass ratio between 11 and 26$/kg are assumed to be

50% Co mattes and 50% Co powder and (iii) all trades with a cost-to-mass ratio above or equal to 26$/kg are assumed to be cobalt powder.

Cobalt intermediates that comprise cobalt mattes covered by the HS code 810520, nickel-cobalt mattes covered by the HS code 750110 and nickel-cobalt sulfide covered by the HS code 750120

The considered HS codes cover cobalt intermediates that are used in EVs but also in other products. Furthermore, the HS code 810520 covers cobalt mattes but also cobalt powder. As stated in the two previous paragraphs, 60% of all batteries are estimated to be used in the electro mobility sector. According to Petavratzi et al.[35], 46% of the global cobalt supply is applied in LIBs that are used in the electro mobility sector. To identify the trade amounts and costs of the Co mattes, the three requirements that are described in the previous paragraph are considered.

Electric vehicle bodies and chassis covered by the HS codes 870710 and 870600, respectively:

The considered HS codes cover car bodies and chassis that are used in EVs but also in other vehicles. According to IEA[39], EVs account for 2.6% of the global car sales. Hence, 2.6% of the sold car bodies and chassis are used in EVs.

Electric vehicle motors covered by the HS codes 850132, 850133, 850152 and 850153:

The considered HS codes cover electric motors that are used in EVs but also in other applications. According to Grand View Research[40], 46% of the globally sold electric motor sales are motors used in EVs.

Electric vehicle wiring covered by the HS code 854430:

The considered HS code covers wiring that is used in EVs but also in other vehicles, aircrafts and ships. Markets and Markets[41], Market Reports World[42] and The Business Research Company[43] state the global market values of wiring used in vehicles, aircraft and ships, respectively. Considering these market values, 78% of the wiring that is covered by the HS code is used in vehicles. According to IEA[39], EVs account for 2.6% of the global car sales. Hence, 2.6% of this wiring is used in EVs.

Aluminium foil covered by the HS code 760719:

The considered HS code covers Al foil that is used in EVs but also in other applications. According to the U.S. Department of Energy[29] and Tsiropoulos et al.[30], 60% of all batteries, in which the Al foil is used, are

LIBs that are applied in the electro mobility sector. Based on the Al foil market shares stated by Grand View Research[44], it is assumed that 6% of the globally supplied Al foil is used in EVs.

Aluminium plate covered by the HS code 760612 and 760692:

The considered HS codes cover Al plates that are used in EVs but also in other applications. Based on information provided by Khoday[45], OECD[46] and Reports and Data[47], it is estimated that 18% of globally supplied Al plates are used for cars. According to IEA[39], EVs account for 2.6% of the global car sales. Hence, 2.6% of these Al plates are used in EVs.

Aluminium wire covered by the HS code 760521and 760529:

The considered HS codes cover Al wires that are used in EVs but also in other applications. Based on information provided by Reports and Data[48] and Grand View Research[49], it is estimated that 15% of globally supplied Al wires are used in cars. According to IEA[39], EVs account for 2.6% of the global car sales. Hence, 2.6% of these Al wires are used in EVs.

Aluminium unwrought covered by the HS codes 760110 and 760120:

The considered HS codes cover Al unwrought that is used in EVs but also in other applications. Based on information provided by Khoday[45], Sauvage[50] and Woodford[51], it is estimated that 28% of globally supplied Al unwrought is used in cars. According to IEA[39], EVs account for 2.6% of the global car sales. Hence, 2.6% of this Al unwrought is used in EVs.

Aluminium oxide covered by the HS code 281820:

The considered HS code covers Al oxide that is used in EVs but also in other applications. Based on information provided by Khoday[45], Sauvage[50] and Woodford[51], it is estimated that 27% of the globally supplied Al oxide is used in cars. According to IEA[39], EVs account for 2.6% of the global car sales. Hence, 2.6% of this Al oxide is used in EVs.

S5. Procedure for the quantification of the supply chain

In this section, the procedure applied for the quantification of the Co and Al supply chain of EVs used in Switzerland is explained by the example of the unit processes and inventory flows described in Figure S1a. Figure S1a illustrates the identified unit processes and related inventory flows for the part of the supply chain concerning EV motor and LIB supply for the EV manufacturing. These unit processes (shown as white squares in Figure S1a) describe processes that are located in specific countries and that use specific materials/products to produce the outputs. The inventory flows (represented by arrows in Figure S1a) constitute flows that describe the outputs of unit processes and the inputs of subsequent supply chain processes (represented by the grey squares in Figure S1a). The inventory flows of each unit process are quantified by following the procedure presented in Figure S1b. This procedure involves the eleven steps illustrated with different colors in Figure S1b. The steps 1-3, which are represented with orange, light blue and yellow colors, are applied to quantify the final product flows. The steps 4-11, which are illustrated with green, red, dark blue and brown colors, are followed to quantify the materials/intermediate product flows upstream of the supply chain.

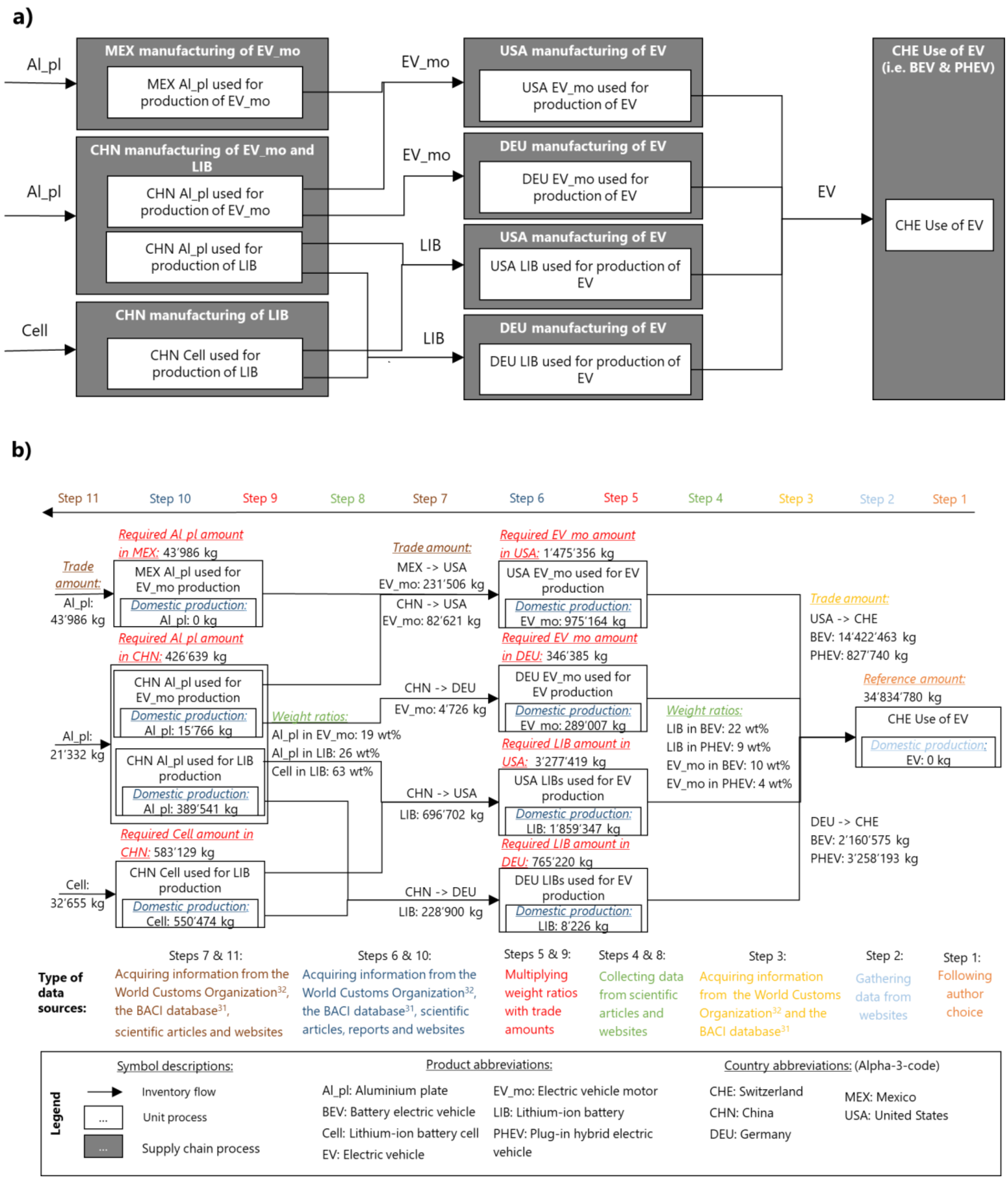

Figure S1: Illustration of the procedure used to quantify the supply chain including (a) the description of unit processes for a part of the cobalt and aluminium supply chain of electric vehicles used in Switzerland in the year 2019 and (b) the exemplary quantification of this part of the supply chain.

The following paragraphs explain the eleven steps for the example introduced in Figure S1b.

- Step 1: the amount of the final product is specified. This amount, which is defined by the authors of this article, corresponds to the Swiss fleet of BEVs and PHEVs in the year 2019.

- Step 2: the domestic production amount of EVs used in Switzerland is determined as 0 kg following Moresi[52].
- Step 3: country-specific trade amounts of these EVs are identified. First, the amounts required from imports are defined by subtracting the Swiss domestic EV production amount from the amount of the Swiss EV fleet. Second, EV amounts that are manufactured in the United States (USA), Germany and other countries and that are exported to Switzerland are determined by selecting related HS codes and applying country-specific trade distributions derived from BACI. For the extraction and formatting of BACI trade data, a python script has been developed within our work. Third, a screening of the EV flows is performed by following the third step of the 'SPOTTER implementation procedure'. Thereby all EV trade amounts that are lower than 1% of the market (i.e. trade and domestic production) are cut-off, which are for example EV flows from Italy to Switzerland with a market share of about 0.08%. Fourth, here-called 'ghost exports' of EVs, i.e. EV exports that are reported in BACI but that do not originate from actual EV production countries, are identified by for example investigating locations stated in company-specific websites. These exports supposedly refer to intermediate trades along the supply chain, which are, for simplicity reasons, neglected in this study. However, amounts of 'ghost exports' still need to be considered to maintain mass balance. Thus, these amounts are reallocated considering existing trade distributions. The trade of ~554 t of BEVs from Austria to Switzerland for example constitutes one of the 'ghost exports'. As the USA contributes by ~57% to the total import of BEVs to Switzerland, ~57% of these 'ghost exports' from Austria is added to the BEV amount traded from the USA to Switzerland.
- Step 4: global average weight ratios of LIBs and EV motors used in BEVs and PHEVs are determined. Due to data availability issues, global instead of country-specific average weight ratios are applied. Following Berjoza and Jurgena[53], the global average weight ratio of for example LIBs used in BEVs is 22%.
- Step 5: amounts of LIBs and EV motors that are required in production countries of EVs used in Switzerland are determined. Thereby traded country-specific EV amounts defined in step 3 are multiplied with weight ratios specified in step 4. To illustrate, the LIB amount of 3'277'419 kg used in

the USA for the production of BEVs and PHEVs traded to Switzerland is calculated as follows: Summing up the product of 14'422'463 kg of BEV and 22 wt% of LIBs used in these BEVs and the product of 827'740 kg of PHEVs and 9 wt% of LIBs used in these PHEVs.

- Step 6: domestic LIB and EV motor production amounts are defined for each country that manufactures EVs used in Switzerland as follows: First, the production amounts of LIBs and EV motors are specified for Germany, the USA and the other EV manufacturing countries. Second, these production amounts and amounts of LIBs and EV motors imports to the EV manufacturing countries are added up to country-specific market volumes of LIBs and EV motors. Third, country-specific production-to-market ratios are multiplied with the required amounts of LIBs or EV motors (see result of step 5) to determine the domestic production amounts. The domestic production amount of 1'859'347 kg of LIBs in the USA for example is calculated by determining the production amount of LIBs in the USA (i.e. 10'637'278 kg) based on studies of, amongst others, Pillot[54], Cerdas Marin et al.,[55] and Mayyas et al.[56] and by considering the import amounts of LIBs to the USA (i.e. 50'704'745 kg) reported in BACI. Due to a lack of country-specific EV motor production amounts in literature, these amounts are estimated by multiplying the global EV motor production amount with country-specific export distributions derived from BACI.
- Step 7: country-specific trade amounts of LIBs and EV motors are determined for the supply chain of EVs used in Switzerland. Analogously to the step 3, these trade amounts are defined by selecting suitable HS codes, considering country-specific trade distributions, applying the cut-off rule and reallocating identified 'ghost exports'. In addition, trade amounts of LIBs and EV motors that are smaller than their minimum amount used in EVs are identified and reallocated. The procedure for reallocation is analogous to the one applied for 'ghost exports'. Such trade amounts may result from the use of global average weight ratios in combination with trade distributions. Finally, the flows of LIBs and EV motors relevant for the assessment over the considered time horizon (i.e. next 5 years) are identified by following the fourth step of the 'SPOTTER implementation procedure'. Thereby, the number of times that LIBs and EV motors are supposedly supplied within the next 5 years is determined based on the lifetimes of these products. This number is then multiplied with the respective

trade amounts. Assuming an eight years lifetime of LIBs[57], a 16 years lifetime of EV motors[58] and a 16 years lifetime of EVs[59], both intermediate products are consequently supplied once over the considered time horizon of 5 years.

All further steps to quantify the supply chain (i.e. steps 8-11 and all following steps) are performed analogously to the steps 4-7.

In contrast to trade amounts of raw materials and products, the trade amounts of the Co and bauxite ores are not defined based on BACI trade data but they are quantified based on production data reported by USGS[60, 61]. The export amounts of the producing countries are thereby estimated based on their production shares on the global production amount. For example, a production share of 69% on the global cobalt production is estimated for the Democratic Republic of the Congo. Thus, 69% of the Co that is imported by the countries within the supply chain is supplied by Congo. Production data reported by USGS instead of BACI trade data are used for defining the ore trade amounts because trade flows of ores may not or only insufficiently be reported in BACI due to traceability issues of artisanal supply chains.[62] On that note, Sun et al.[63] have highlighted that trade flows of Co ores from Congo, the country extracting the highest amounts of Co worldwide, have not been included in the trade data reported by BACI.

S6. Calculation of bottleneck scores and characterization factors

As explained in section 3.3 of the main article, the bottleneck scores are calculated by multiplying the inventory flow amounts of the individual materials/products with the respective characterization factors (CFs). These CFs represent the cause-effect chains between the considered events and impacts of supply disruptions. Figure S2 shows the thus analyzed events and impacts exemplarily for the extract of the supply chain comprising the flow of Co powder from China to the Republic of Korea.

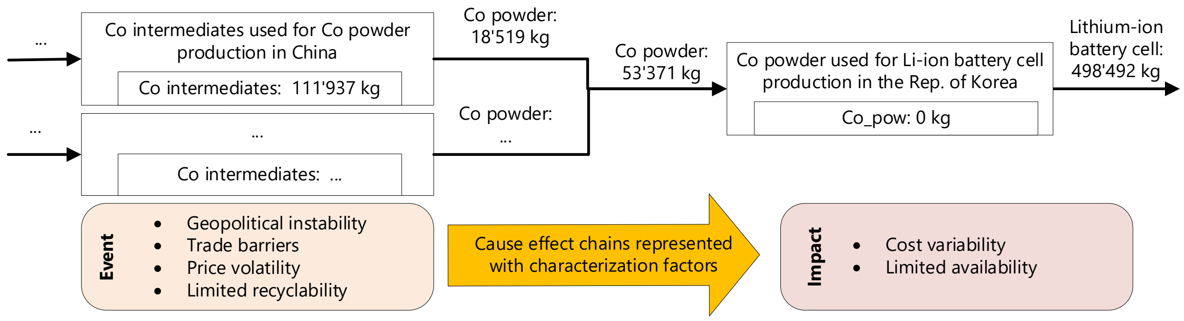

Figure S2: Outline of considered cause-effect chains along the supply chain illustrated for the example of supply disruption events and impacts for the cobalt (Co) powder flow from China to the Republic of Korea

Just as demonstrated in Figure S2, CFs are applied to represent cause-effect chains between supply disruption events and impacts for each unit process along the Co and Al supply chains of EVs used in Switzerland.

As shown by Berr et al.[64], the applied CFs are calculated individually for each event and each impact by defining differently the four types of indicators that are described in Equation (2) of the main article. Table S2 illustrates how these indicators are defined and quantified in the different cases exemplarily for the Co powder flow described in Figure S2.

Table S2: Quantification of indicators used to calculate the characterization factors presented exemplarily for the cobalt powder (Co_pow) flow illustrated in Figure S2. Because indicators for economic resource depletion and child labor restrictions are not described in the example given in Figure S2, these indicators are quantified based on cobalt resource (Co_res) extraction in the Democratic Republic of the Congo (COD) and cobalt ore (Co_ore) flows from Congo, respectively.

a) Indicators for supply disruption events (EIs)

Indicator		Definition and quantification	Data sources
Indicators for country-specific events	Indicator for depletion of economic resource (ERD)	$ERD_{Co\_res,COD} = \frac{extr_{Co\_res,COD} - repl_{Co\_res,COD}}{stock_{Co\_res,COD}} = \frac{100'000\ t - 0\ t}{3'600'000\ t}$	Extraction rates (extr.) and replenishment rates (repl.) of Co_res are acquired from USGS[60]. Replenishment rates of metallic resources are assumed to be 0 t over the considered time horizon (i.e. next 5 years).
	Indicator for geo-political instability (GI)	$GI_{Co\_pow,CHN} = \frac{100 - WGI(PS)_{CHN}}{100} = \frac{100 - 38.10}{100}$	Values for the Worldwide Governance Indicator (Political Stability and Absence of Violence/Terrorism) (WGI(PS)) are acquired from the World Bank[65].
	Indicator for child labor restrictions (CLR)	$CLR_{Co\_ore,COD} = (CLI_{Co\_pow,COD} * 0.2) = (4 * 0.2)$	Values for the indicator of child labor risk (CLI) in the sector of mining of metal ore (i.e. the sector related to Co_ore production in the Social Hotspot Database) are acquired from Benoit Norris et al.[66]
	Indicator for trade barriers (TB)	$TB_{Co\_pow,CHN} = \frac{100 - TABI_{CHN}}{100} = \frac{100 - 86.5}{100}$	Values for the Trading Across Borders Indicator (TABI) are acquired from the World Bank[67].
Indicators for global events	Indicator for price volatility (PV)	$market\ price_{Co\_pow} = \frac{global\ cost_{Co\_pow}}{global\ mass_{Co\_pow}}$ $PV_{Co\_pow} = \frac{highest\ market\ price_{Co\_pow}\ (last\ 3\ years)}{lowest\ market\ price_{Co\_pow}\ (last\ 3\ years)} = \frac{376\ ^{\$}/_{kg}}{89\ ^{\$}/_{kg}}$	Global costs and mass of Co_pow flows over the last three years are acquired from BACI[24].
	Indicator for limited recyclability (LR)	$LR_{Co\_pow} = 100\% - EoLRR_{Co\_pow} = 100\% - 32\%$	The End-of-Life recycling rate (EoLRR) of Co_pow used in LIBs is acquired from Church and Wuennenberg[68]. EoLRR of Al is 90% according to The Aluminium Association[69].

b) Indicators for supply disruption event period (t)

Indicator	Definition and quantification	Data sources
Indicator for event duration (t)	t = 5 years	t is assumed equal to the considered time horizon, unless specific information about the expected event duration is available.

c) Indicators for supply diversity (DIs)

Indicator	Definition and quantification	Data sources
Indicator for resource concentration	$DI_{Co\_res,COD} = 100\%$	Resource concentrations of 100% are considered in the SPOTTER approach (see explanations in Berr et al.[64])
Indicator for market concentration (used in combination with indicators for country-specific events)	$weight\%(Co\_pow\ in\ EV) = 28\%$ $market_{Co\_pow,KOR} = production_{Co\_pow,KOR} + import_{Co\_pow,KOR}$ $DI_{Co\_pow,CHN,KOR} = \frac{import_{Co\_pow,CHN,KOR}}{market_{Co\_pow,KOR}} = \frac{121'994\ kg}{0\ kg\ +\ 1'999'538\ kg}$	Co_pow import amounts are acquired from BACI[24] and Co_pow production amounts are acquired from USGS[60]. These import and production amounts are multiplied with the weight percentage (weight%) of Co_pow used in EVs, which is estimated based on data from Tsiropoulos et al.[30] and Petavratzi et al.[35]
Indicator for production concentration (used in combination with indicators for global events)	$HHI_{Co\_pow} = \left(\frac{production_{Co\_pow,CHN}}{production_{Co\_pow,global}}\right)^2 + \sum_n \left(\frac{production_{Co\_pow,n}}{production_{Co\_pow,global}}\right)^2$ $DI_{Co\_pow} = normalized\ HHI_{Co\_pow} = \frac{(HHI_{Co\_pow}) * 1/n}{1 - 1/n} = \frac{0.48 * 1/15}{1 - 1/15}$	Co_pow production amounts and the number of production countries (n) are acquired from USGS[60].

d) Indicators for vulnerability to physical shortage (PVI)

Indicator	Definition and quantification	Data sources
Indicator for vulnerability to physical shortage (PVI)	$weight\%(Co\_pow\ in\ EV) = 28\%$<br>$weight\%(Co\ in\ Co\_pow) = 100\%$<br><br>The in-use stock of Co_pow is calculated by adding up the past production amounts of Co_pow used in LIBs over the LIB lifetime.<br><br>$PVI_{Co\_pow} = \frac{production_{Co\_pow}}{\left(in\_use\ stock_{Co\_pow}\right)^2} = \frac{36'475'332\ \frac{kg}{year}}{(123'711'460\ kg)^2}$	Current and past Co_pow and Co_ore production amounts are acquired from USGS[60, 70-72]. Co_pow production amounts are multiplied with the weight% that is estimated in Table S2c. The lifetime of LIBs, in which Co_pow are used, is defined using data from Argue[57].

e) Indicator for economic importance or economic damage (EVI)

Indicator	Definition and quantification	Data sources
Indicator for economic importance (used for analysis of cost variability (CV))	The costs of Co_pow in the supply chain ($cost_{Co\_pow,supply\ chain}$) is calculated by the sum of the following two factors (i) all Co_pow flow amounts (e.g. $m_{Co\_pow,Cell,CHN,KOR}$) multiplied with their respective cost-to-mass ratio (e.g. $\frac{cost_{Co\_pow,CHN,KOR}}{mass_{Co\_pow,CHN,KOR}}$) and (ii) the domestic production amounts (e.g. $production_{Co\_pow,KOR}$) multiplied with the global cost-to-mass ratio (i.e. $\frac{cost_{Co\_pow,global}}{mass_{Co\_pow,global}}$) $$EVI(CV)_{Co\_pow,Cell,CHN,KOR} = \frac{m_{Co\_pow,Cell,CHN,KOR} * \frac{cost_{Co\_pow,CHN,KOR}}{mass_{Co\_pow,CHN,KOR}}}{cost_{Co\_pow,supply\ chain}} = \frac{18'519\ kg * \frac{4'106'066\ \$}{99'392\ kg}}{3'954'741\ \$}$$	The Co_pow and Cell flows as well as the domestic production amounts and trade amounts of Co_pow are determined following the procedure described in section 2.3 of the main article. Data for the cost-to-mass ratios of Co_pow and Cells are acquired from BACI[24].
Indicator for economic damage (used for analysis of limited availability (LA))	The revenue of Cells affected by supply disruptions of Co_pow is determined by the cost of Cells traded from KOR to any other country i. This cost is additionally multiplied with 1 minus the share of revenue that originates from domestic production, when events are analyzed for which domestic production is considered risk-free (i.e. geopolitical instability, trade barriers and child labor restrictions). The cost of Cells in the supply chain (i.e. ($cost_{Cell,supply\ chain}$) is calculated analogously to $cost_{Co\_pow,supply\ chain}$. $$EVI(LA)_{Cell,KOR} = \frac{\sum_i \left(m_{Cell,KOR,i} * \frac{cost_{Cell,KOR,i}}{mass_{Cell,KOR,i}}\right) * \left(1 - \frac{production_{Co\_pow,Cell,KOR}}{market_{Co\_pow,Cell,KOR}}\right)}{cost_{Cell,supply\ chain}} = \frac{21'603'519\ \$ * 100\%}{50'385'459\ \$}$$	

Material/product abbreviations: Cell: Cell of lithium-ion traction battery, Co_ore: Cobalt ore, Co_pow: Cobalt powder, Co_res: Cobalt resource, EV: electric vehicle, LIB: Lithium-ion traction battery; country abbreviations: COD: Democratic Republic of the Congo, CHN: China, KOR: Republic of Korea

The indicators that are required for the calculation of all other CFs are defined analogously to the examples given in Table S2. Respective indicator data and data sources are provided in the following Excel sheets: "LCIA data LIB & EV.xlsx", "LCIA data EV body, motor, chassis & wiring.xlsx", "LCIA data Al foil, plate & wire.xlsx", "LCIA data Co mining & processing.xlsx", "LCIA data Al ore, oxide & unwrought.xlsx", "Geopolitical instability indicator.xlsx", "Trading_Across_Borders_Indicator_score.xlsx", "Price volatility indicator.xlsx" and "Depletion potential indicator.xlsx". Note that the data to quantify the indicator for child labor restrictions can only be made available on condition that a suitable license for the Social Hotspot Database[66] has been purchased.

Furthermore, as explained by Berr et al.[64], two important issues regarding the indicator definitions are: On the one hand, the *DI* values need to be defined differently in case of country-specific and global events. Definitions for both types of events are provided by Bach et al.[73] and Berr et al.[64] On the other hand, the *PI* and *EVI* values need to be newly defined for each case study, as their equations involve case-specific scaling or case-specific elements (i.e. $m_{Co\_pow,Cell,CHN,KOR}$, $m_{Co\_ore,COD}$, $cost_{Cell,supply\ chain}$ and $cost_{Co\_ore,supply\ chain}$). Further explanations concerning these two issues can be found in Berr et al.[64]

After all EIs, DIs, PVIs and EVIs have been quantified, the individual bottleneck scores are calculated. Equation (S1) demonstrates the calculation of a bottleneck score for an example shown in Figure S2, where cost variability (CV) of a Co powder (Co_pow) flow is caused by geopolitical instability (GI) in China (CHN) and affects the LIB cell (Cell) production in the Republic of Korea (KOR). The related inventory flow amount is defined in the Excel sheet "Inventory flows_Swiss_EV.xlsx" provided in the SI and the required indicator values are presented in Table S3.

$$\begin{aligned} CV\ score(GI)_{Co\_pow,Cell,CHN,KOR} &= \left(m_{Co\_pow,Cell,CHN,KOR}\right) * \left(GI_{Co\_pow,CHN} * t\right) * \\ &* \left(DI_{Co\_pow,CHN,KOR}\right) * \left(PVI_{Co\_pow}\right) * \left(EVI(CV)_{Co\_pow,Cell,CHN,KOR}\right) = \\ &\left(m_{Co\_pow,Cell,CHN,KOR}\right) * \left(\frac{100 - WGI(PS)_{CHN}}{100} * t\right) * \left(\frac{import_{Co\_pow,CHN,KOR}}{market_{Co\_pow,KOR}}\right) \\ &* \left(\frac{production_{Co\_pow}}{\left(in - use\ stock_{Co\_pow}\right)^2}\right) * \left(\frac{m_{Co\_pow,Cell,CHN,KOR} * \frac{cost_{Co\_pow,CHN,KOR}}{mass_{Co\_pow,CHN,KOR}}}{cost_{Co\_pow,supply\ chain}}\right) \\ &\approx 1.6 * 10^{-6} \end{aligned} \qquad (S1)$$

S7. Relative magnitude of hotspots for each impact type

To define the relative magnitude of hotspots, individual hotspot scores calculated for each of the impact types following Equation **Error! Reference source not found.** in the main article are divided by the overall impact scores listed in Table S3.

Table S3: Overall impact scores for the considered impact types calculated following Equation **Error! Reference source not found.** in the main article. For illustration purposes, the presented bottleneck scores are multiplied by a factor of 1000.

Impact category	Supply disruption events	Impact score [dimensionless]
**Cost variability**	Geopolitical instability	0.630
	Trade barriers	0.289
	Child labor restrictions	0.170
	Price volatility	1.475
	Limited recyclability	0.430
	**Total of all events**	**2.994**
**Limited availability**	Geopolitical instability	1.177
	Trade barriers	0.484
	Child labor restrictions	0.265
	Price volatility	1.312
	Limited recyclability	0.360
	Resource depletion	0.208
	**Total of all events**	**2.807**

The relative magnitudes of hotspots are presented on the level of the impact category (Figure S3) and on the level of the impact category and supply disruption event (Figure S4).

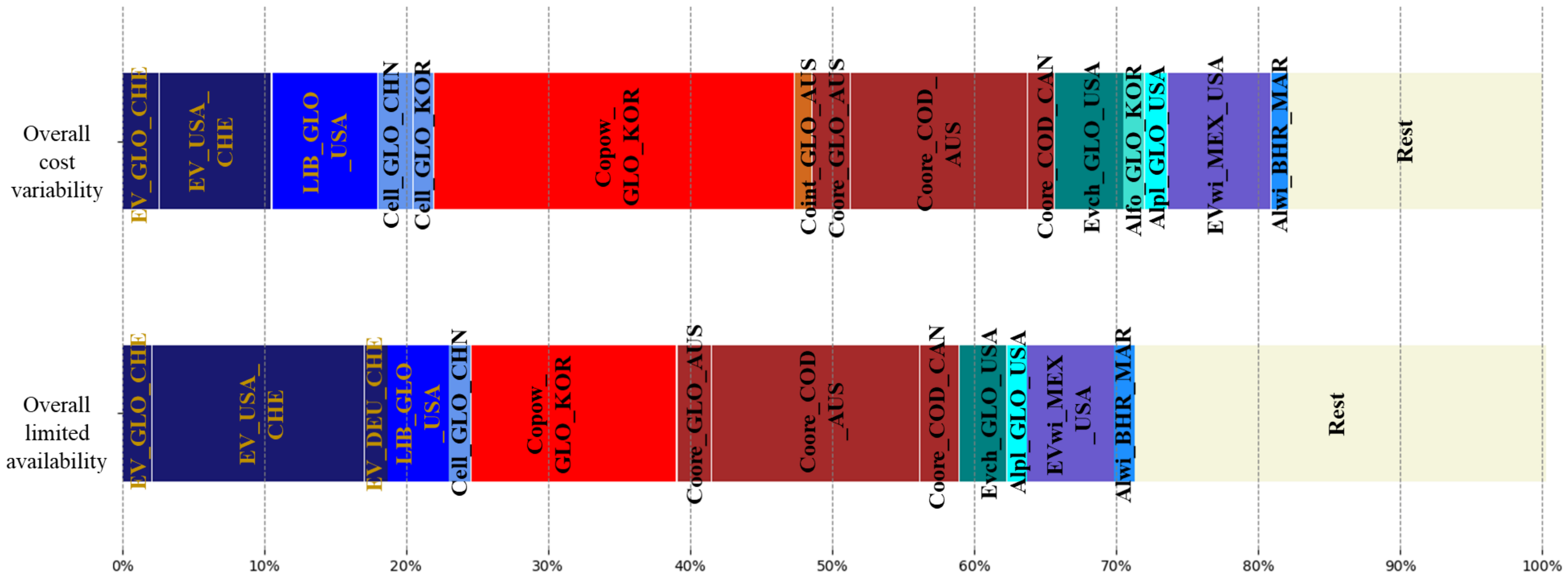

Figure S3: Magnitude of hotspots per impact category considering the cost variability and the limited availability related to material/product flows. Abbreviations for the materials/products are explained in Figure 2 of the main article and abbreviations for countries are based on alpha-3 codes (GLO refers to flows originating from the global market).

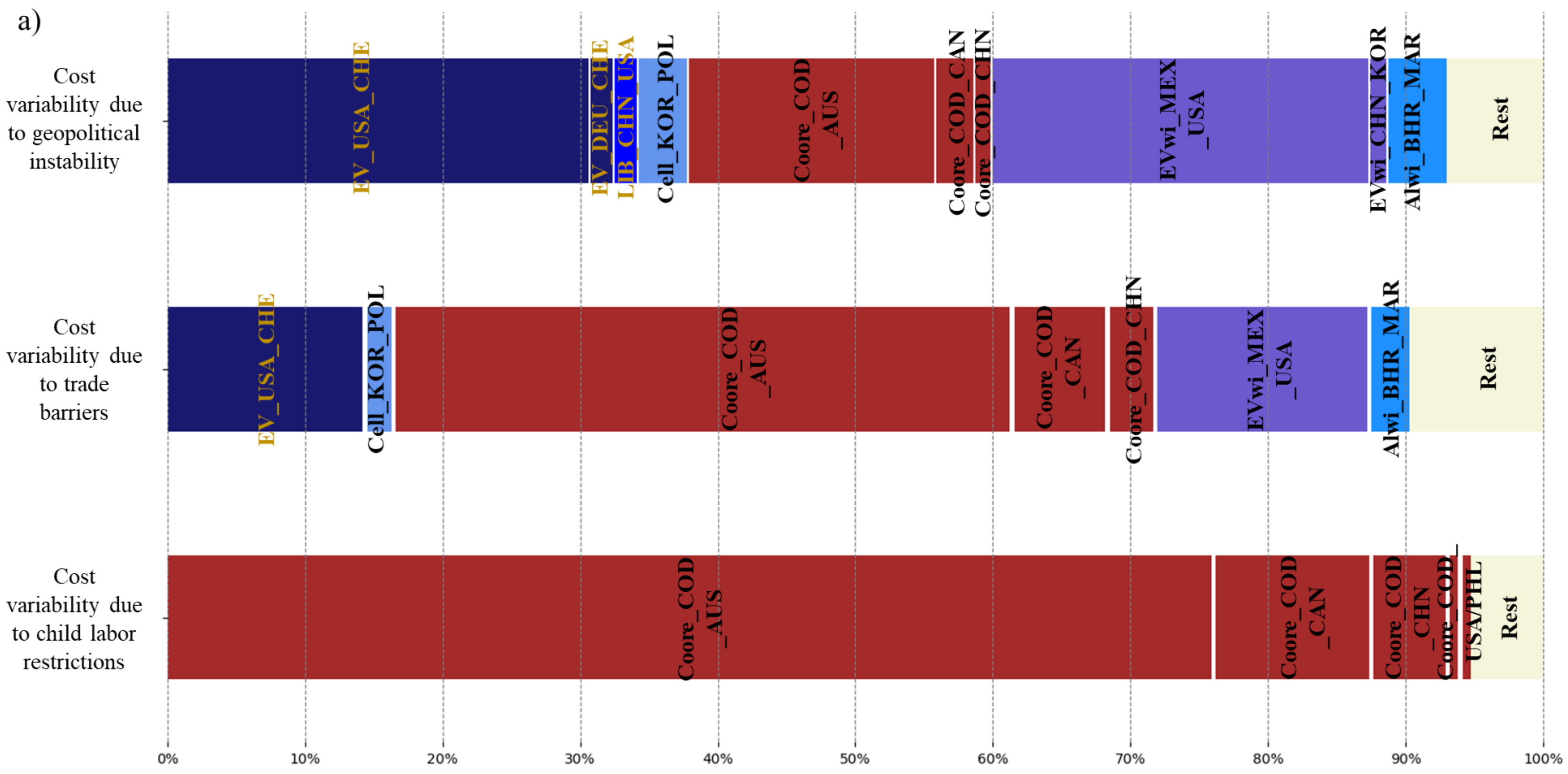

Figure S4: Magnitude of hotspots per event and impact category considering a) the cost variability and b) the limited availability caused by geopolitical instability, trade barriers and child labor restrictions as well as c) the cost variability and d) the limited availability caused by price volatility, limited recyclability and economic resource depletion. Abbreviations for the materials/products are explained in Figure 4 of the main article and abbreviations for countries are based on alpha-3 codes (GLO refers to flows originating from the global market).

b)

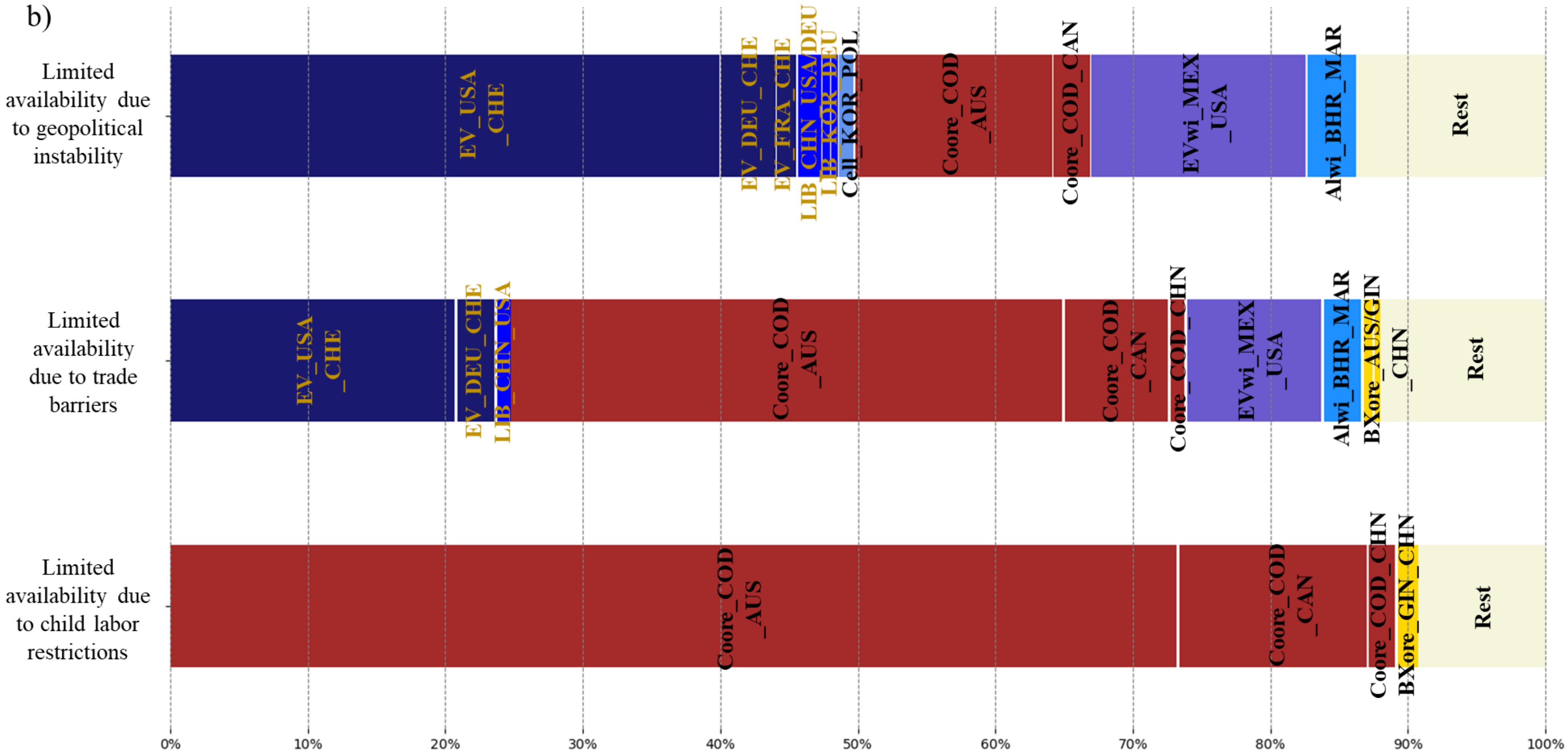

Figure S4 (continued)

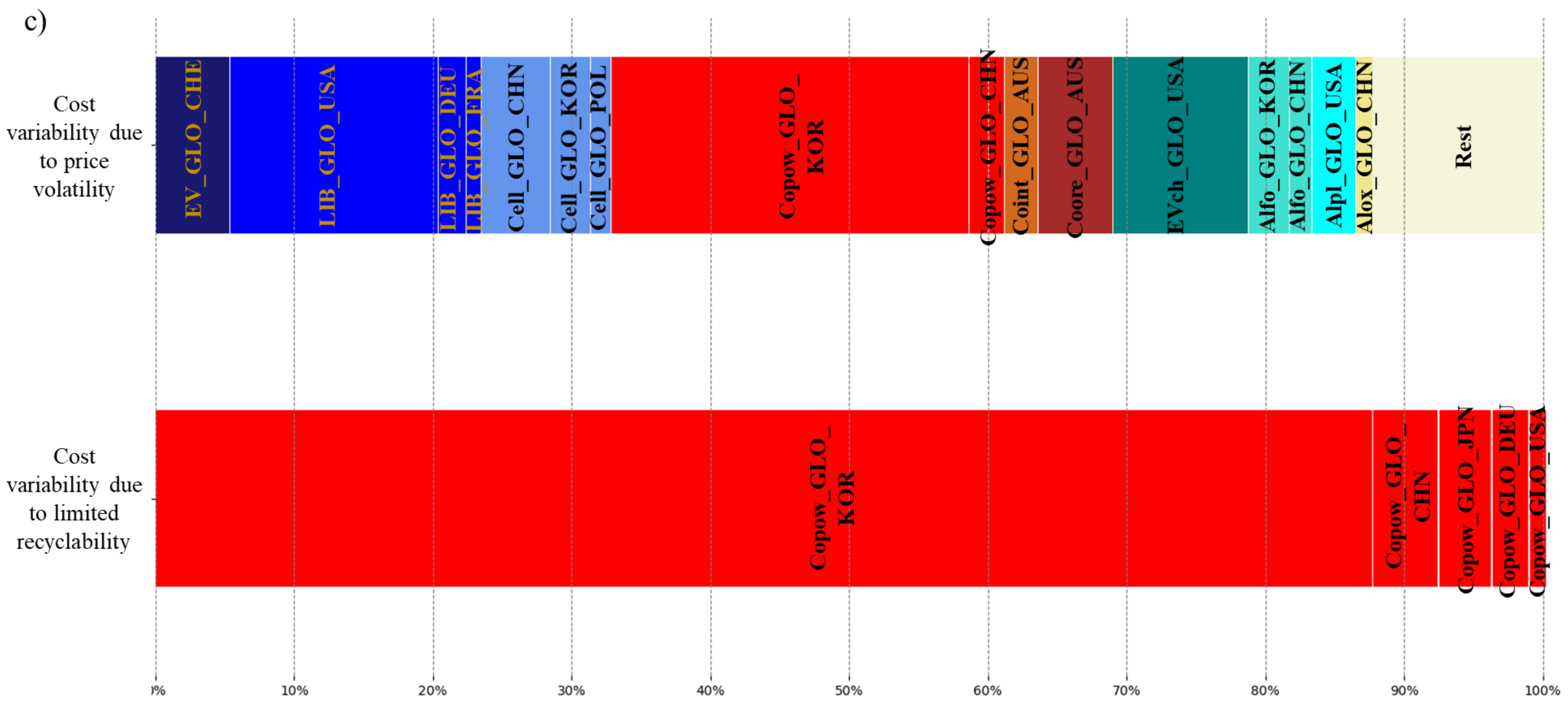

Figure S4 (continued)

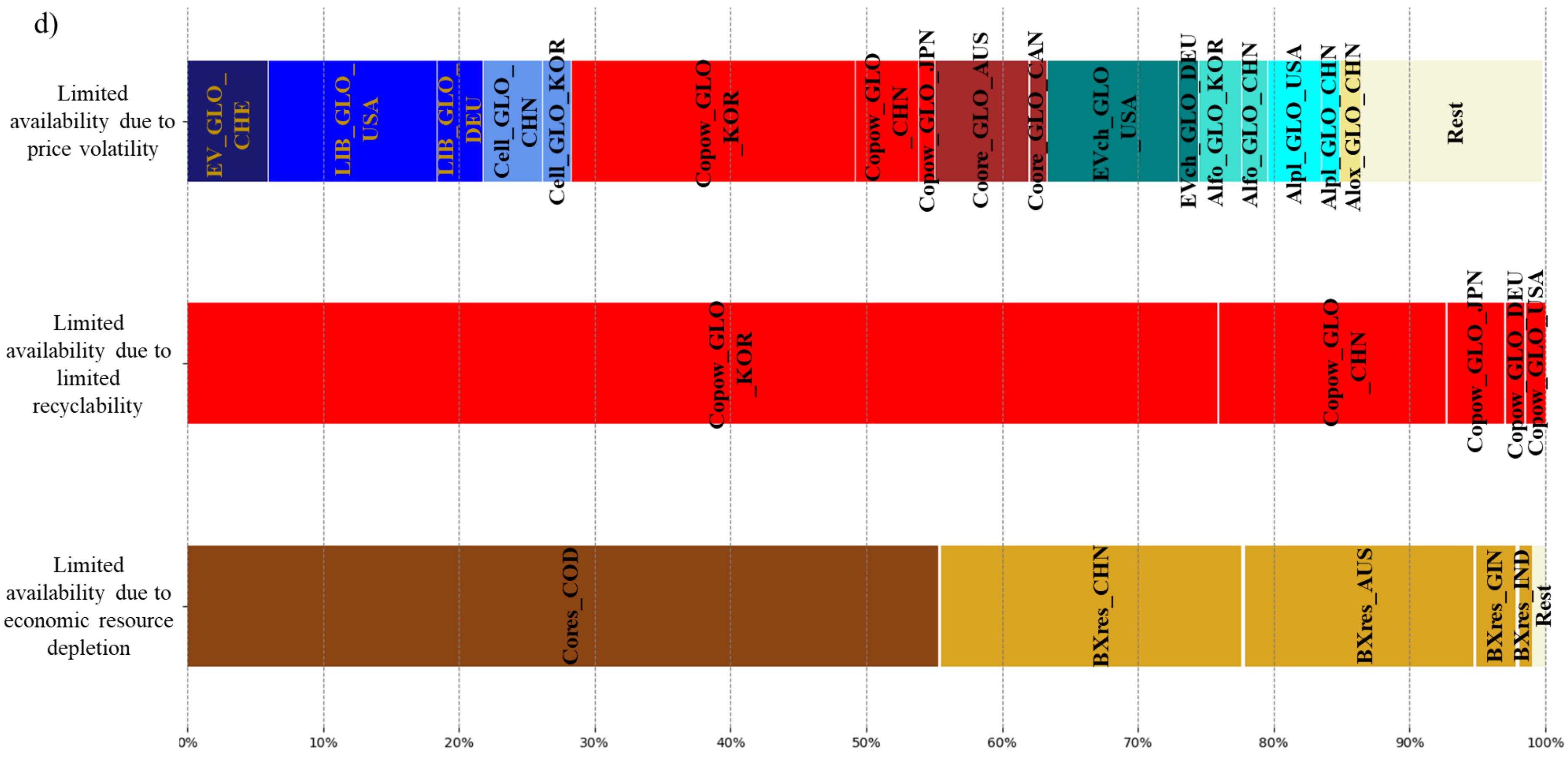

Figure S4 (continued)

S8. Comparison with existing studies

The studies performed by Cimprich et al.[74, 75] and Lütkehaus et al.[76] have already analyzed supply disruption hotspots for EVs using criticality assessment approaches integrated into the Life Cycle Sustainability Assessment (LCSA) framework. While their studies focus particularly on the supply of different raw materials, our study evaluates the full Co and Al supply chains of EVs. Furthermore, in contrast to our study, the supply of cobalt is not considered in their supply chain analysis. Finally, our study represents the country-specific variabilities of impacts on flows along the supply chain, while their studies do not illustrate such variabilities but only describe the impact on the supply of the specific raw material. For example, the hotspots for cobalt ore supplied from Congo to Australia and cobalt ore supplied from Congo to Canada (see Figure 4a-f in the main article) could thus not have been identified in their studies. Consequently, the results of the studies conducted by Cimprich et al.[74, 75] and Lütkehaus et al.[76] are hardly comparable with the results of our study.

Supply bottlenecks (i.e. supply disruption hotspots) along EV supply chains have also been identified by studies performing criticality assessments outside of the LCSA framework. These studies inevitably lack the benefits of assessments within this framework that are described in section 1 of the main article. Examples are studies conducted by Moss et al.[77] and Blagoeva et al.[78], which just as we did in our study (see Figure 2 and Figure 4 in the main article), highlight an instability of the cobalt supply. However, in contrast to our study, these two studies only analyze the raw materials supply. The EU Foresight Study developed by Bobba et al.[79] in turn has identified supply bottlenecks at several stages of the supply chain. This study considers, amongst others, supply chains of batteries and traction motors. The EU Foresight Study as well as our study have for example identified comparatively big supply bottlenecks associated with LIBs and LIB cells exported by China and other Asian countries. However, several other supply disruption hotspots that have been identified in our study do not occur in the EU Foresight Study. Examples are the supply disruption hotspots defined by impacts related to Al wire, EV wiring and bauxite ore supply (see Figure 4a-f in the main article). Reasons for the identification of additional hotspots in our study may be: on the one hand, the SPOTTER approach used in our study allows for identifying

impacts related to material/product flows between specific countries along the global supply chains of Switzerland, while the approach applied for the EU Foresight Study allows for identifying supply bottlenecks related to the EU imports of materials/products. On the other hand, our study represents bottleneck scores for the individual materials/products, while the EU Foresight Study presents aggregated bottleneck scores encompassing scores for all considered raw materials, processed materials, components or assemblies, respectively.

References:

(1) Irle, R. *EV Volumes.com The Electric Vehicle World Sales Database*. 2021. https://www.ev-volumes.com/ (accessed 25/08/2021).
(2) Hirschberg, S.; Bauer, C.; Cox, B.; Heck, T.; Hofer, J.; Schenler, W.; Simons, A.; Del Duce, A.; Althaus, H.-J.; Georges, G.; Krause, T.; Gonzalez Vaya, M.; Ciari, F.; Waraich, R.; Jäggi, B.; Stahel, A.; Froemelt, A. *Opportunities and challenges for electric mobility: an interdisciplinary assessment of passenger vehicles*; 2016.
(3) Lattanzio, R. K. *Environmental Effects of Battery Electric and Internal Combustion Engine Vehicles*; 2020.
(4) Castelvecchi, D. *Electric cars and batteries: how will the world produce enough?* 2021. https://www.nature.com/articles/d41586-021-02222-1 (accessed 21/10/2021).
(5) Rajeev Kumar, A. *Focus on Expansion of Hydrogen and Electric Fleets for Passenger and Freight Transport in United Kingdom*; 2016.
(6) Egede, P. Electric Vehicles, Lightweight Design and Environmental Impacts. In *Environmental Assessment of Lightweight Electric Vehicles*, Egede, P. Ed.; Springer International Publishing, 2017; pp 9-40.
(7) PrimecomTech. *What Types Of Motors Are Used In Electric Vehicles?* 2019. https://www.primecom.tech/blogs/news/what-types-of-motors-are-used-in-electric-vehicles (accessed 22/10/2021).
(8) Schröder, M. *Electric Vehicle and Electric Vehicle Component Production in Thailand*; ERIA Research Project Report FY2021 no.03, Jakarta: ERIA, 2021.
(9) Matt, D.; Boubaker, N.; Aitakkache, M.; Enrici, P.; Huselstein, J.; Martire, T. *High Power Very Low Voltage Electric Motor for Electric Vehicle*; 2021. DOI: https://doi.org/10.5772/intechopen.99134.
(10) Sullivan, J.; Kelly, J.; Elgowainy, A. *Vehicle Materials: Material Composition of Powertrain Systems*; 2015.
(11) International Driving Authority. *Car paints: composition and effects of painting*. 2019. https://idaoffice.org/posts/car-paints-composition-and-effects-of-painting/ (accessed 02/09/2021).
(12) Perner, A.; Vetter, J. 8 - Lithium-ion batteries for hybrid electric vehicles and battery electric vehicles. In *Advances in Battery Technologies for Electric Vehicles*, Scrosati, B., Garche, J., Tillmetz, W. Eds.; Woodhead Publishing, 2015; pp 173-190.
(13) Diekmann, J.; Hanisch, C.; Froböse, L.; Schälicke, G.; Loellhoeffel, T.; Fölster, A.-S.; Kwade, A. Ecological Recycling of Lithium-Ion Batteries from Electric Vehicles with Focus on Mechanical Processes. *Journal of The Electrochemical Society* **2016**, *164* (1), A6184-A6191. DOI: https://doi.org/10.1149/2.0271701jes.
(14) Coffin, D.; Horowitz, J. The Supply Chain for Electric Vehicle Batteries. *Journal of International Commerce and Economics* **2018**.
(15) Liu, M.; Guo, Y.; Wang, J.; Yergin, M. Corrosion avoidance in lightweight materials for automotive applications. *npj Materials Degradation* **2018**, *2* (1), 24. DOI: https://doi.org/10.1038/s41529-018-0045-2.
(16) Tsirogiannis, E. Design of an efficient and lightweight chassis, suitable for an electric car. 2015.
(17) Rassõlkin, A.; Belahcen, A.; Kallaste, A.; Vaimann, T.; Lukichev, D.; Orlova, S.; Heidari, H.; Asad, B.; Pando-Acedo, J. Life cycle analysis of electrical motor-drive system based on electrical machine type. *Proceedings of the Estonian Academy of Sciences* **2020**, *69*, 162–177. DOI: https://doi.org/10.3176/proc.2020.2.07.
(18) Yu, M. Aluminium cables in automotive applications PRESTUDY OF ALUMINIUM CABLE USES IN SCANIA PRODUCTS & FAILURE ANALYSIS AND EVALUATION. 2016.
(19) Baars, J.; Domenech, T.; Bleischwitz, R.; Melin, H. E.; Heidrich, O. Circular economy strategies for electric vehicle batteries reduce reliance on raw materials. *Nature Sustainability* **2021**, *4* (1), 71-79. DOI: https://doi.org/10.1038/s41893-020-00607-0.
(20) Schmidt, T.; Buchert, M.; Schebek, L. Investigation of the primary production routes of nickel and cobalt products used for Li-ion batteries. *Resources, Conservation and Recycling* **2016**, *112*, 107-122. DOI: https://doi.org/10.1016/j.resconrec.2016.04.017.

(21) Liu, G.; Muller, D. B. Mapping the global journey of anthropogenic aluminum: a trade-linked multilevel material flow analysis. *Environ Sci Technol* **2013**, *47* (20), 11873-11881. DOI: https://doi.org/10.1021/es4024404.
(22) Liu, G.; Bangs, C. E.; Müller, D. B. Stock dynamics and emission pathways of the global aluminium cycle. *Nature Climate Change* **2012**, *3* (4), 338-342. DOI: https://doi.org/10.1038/nclimate1698.
(23) Wernet, G.; Bauer, C.; Steubing, B.; Reinhard, J.; Moreno-Ruiz, E.; Weidema, B. The ecoinvent database version 3 (part I): overview and methodology. *The International Journal of Life Cycle Assessment* **2016**, *21* (9), 1218-1230, journal article. DOI: https://doi.org/10.1007/s11367-016-1087-8.
(24) Gaulier, G.; Zignago, S. *BACI: International Trade Database at the Product-Level. The 1994-2007 Version*; CEPII, 2010. http://www.cepii.fr/CEPII/fr/publications/wp/abstract.asp?NoDoc=2726 (accessed 10/06/2021).
(25) World Customs Organization. *Harmonized System*. 2021. https://www.wcotradetools.org/en/harmonized-system/2017/en/ (accessed 02/09/2021).
(26) Reister, M.; Muryawan, M. *Quantity and Weight Data in UN Comtrade - Standard Unit Values*. 2019. https://unstats.un.org/wiki/display/comtrade/Quantity+and+Weight+Data+in+UN+Comtrade (accessed 17/03/2023).
(27) Godoy León, M. F.; Blengini, G. A.; Dewulf, J. Analysis of long-term statistical data of cobalt flows in the EU. *Resources, Conservation and Recycling* **2021**, *173*, 105690. DOI: https://doi.org/10.1016/j.resconrec.2021.105690.
(28) European Commission. *Study on the review of the list of Critical Raw Materials - Critical Raw Materials Factsheets*; 2017.
(29) U.S. Department of Energy. *Energy Storage Grand Challenge: Energy Storage Market Report*; 2020.
(30) Tsiropoulos, I.; Tarvydas, D.; Lebedeva, N. *Li-ion batteries for mobility and stationary storage applications - Scenarios for costs and market growth*; 2018.
(31) Grand View Research. *Electric Vehicle Battery Market Size, Share & Trends Analysis Report By Battery Type (Lithium-ion, Lead-acid, Nickel-metal Hydride, Sodium-ion), By Vehicle Type, By Propulsion, By Region, And Segments Forecasts, 2019 - 2025*; 2019. https://www.grandviewresearch.com/industry-analysis/electric-vehicle-battery-market (accessed 20/09/2021).
(32) Wong, Y. S.; Chan, C. C. Vehicle Energy Storage: Batteries. In *Encyclopedia of Sustainability Science and Technology*, Meyers, R. A. Ed.; Springer New York, 2012; pp 11502-11522.
(33) Mongird, K.; Fotedar, V.; Viswanathan, V.; Koritarov, V.; Balducci, P.; Hadjerioua, B.; Alam, J. *Energy Storage Technology and Cost Characterization Report*; Pacific Northwest National Laboratory, Argonne National Laboratory, Oak Ridge National Laboratory, 2019.
(34) BloombergNEF. *Battery Pack Prices Cited Below $100/kWh for the First Time in 2020, While Market Average Sits at $137/kWh*. 2020. https://about.bnef.com/blog/battery-pack-prices-cited-below-100-kwh-for-the-first-time-in-2020-while-market-average-sits-at-137-kwh/ (accessed 21/09/2021).
(35) Petavratzi, E.; Gunn, G.; Kresse, C. *BGS COMMODITY REVIEW: Cobalt*; 2019.
(36) London Metal Exchange. *Cobalt*. 2021. https://tradingeconomics.com/commodity/cobalt (accessed 25/08/2021).
(37) Trading Economics. *Cobalt*. 2021. https://tradingeconomics.com/commodity/cobalt (accessed 21/10/2021).
(38) European Commission. *Study on the EU's list of Critical Raw Materials (2020) - Critical Raw Materials Factsheet (Final)*; Brussels, 2020.
(39) IEA. *Global EV Outlook 2020*; Paris, 2020. https://www.iea.org/reports/global-ev-outlook-2020.
(40) Grand View Research. *Electric Motor Sales Market Size, Share & Trends Analysis Report By Application, By Power Output (Integral HP Output, Fractional HP Output), By Motor Type (Hermetic, AC, DC), By Region, And Segment Forecasts, 2021 - 2028*. 2021. https://www.grandviewresearch.com/industry-analysis/electric-motor-market (accessed 11/10/2021).
(41) Markets and Markets. *Automotive Wiring Harness Market*. 2019. https://www.marketsandmarkets.com/Market-Reports/automotive-wiring-harness-market-170344950.html (accessed 29/10/2021).
(42) Market Reports World. *Aircraft Wire and Cable Market Share, Size,Growth Global Leading Players, Industry Updates, Future Business Prospects, Forthcoming Developments and Future Investments by*

Forecast to 2027. 2021. https://www.yournewsnet.com/story/44193521/aircraft-wire-and-cable-market-share-sizegrowth-global-leading-players-industry-updates-future-business-prospects-forthcoming-developments-and-future (accessed 29/10/2021).
(43) The Business Research Company. *Shipboard Cables Global Market Report 2020*. 2020. https://www.thebusinessresearchcompany.com/report/shipboard-cables-global-market-report-2020-30-covid-19-impact-and-recovery (accessed 29/10/2021).
(44) Grand View Research. *Aluminum Foil Market Size, Share & Trends Analysis Report By End-use, By Application, By Region, And Segment Forecasts, 2021 - 2028*. 2021. https://www.grandviewresearch.com/industry-analysis/aluminum-foil-market (accessed 15/11/2021).
(45) Khoday, T. *Aluminium - Base Metals*. 2019. https://fyers.in/school-of-stocks/chapter/commodities/aluminium.html (accessed 10/11/2021).
(46) OECD. *Materials Case Study 2: Aluminium*; Belgium, 2010. https://www.oecd.org/environment/waste/46194971.pdf.
(47) Reports and Data. *Aluminum Alloys Market Size, Share And Industry Analysis By End-Use*. 2020. https://www.reportsanddata.com/report-detail/aluminum-alloys-market (accessed 10/11/2021).
(48) Reports and Data. *Aluminium Wire Market*. 2020. https://www.reportsanddata.com/report-detail/aluminum-wire-market (accessed 29/10/2021).
(49) Grand View Research. *Magnet Wire Market Size, Share & Trends Analysis Report By Material, By Product, By End Use, By Region, And Segment Forecasts, 2019 - 2025*. 2019. https://www.grandviewresearch.com/industry-analysis/magnet-wire-market (accessed 16/11/2021).
(50) Sauvage, J. *Measuring distortions in international markets - The aluminium value chain*; 2019. https://www.oecd.org/officialdocuments/publicdisplaydocumentpdf/?cote=TAD/TC(2018)5/FINAL&docLanguage=En.
(51) Woodford, C. *Aluminium*. 2021. https://www.explainthatstuff.com/aluminum.html (accessed 22/11/2021).
(52) Moresi, R. *The Swiss Automotive Industry Is Not Known For Producing Complete Cars; They Supply Vehicle Parts, Accessories, And More.* 2018. http://www.svbgf.ch/portrait/mitgliederverzeichnis/anbieterinnen/Swiss-automotive-industry/not-known-for-complete-cars/supplies-parts-accessories (accessed 22/10/2020).
(53) Berjoza, D.; Jurgena, I. Effects of change in the weight of electric vehicles on their performance characteristics. *Agronomy Research* **2017**, *15*, 952-963.
(54) Pillot, C. *EU battery demand and supply (2019-2030) in a global context*; 2020. https://www.eurobat.org/images/Avicenne_EU_Market_-_summary_110321.pdf.
(55) Cerdas Marin, J. F.; Titscher, P.; von Drachenfels, N.; Schmuch, R.; Winter, M.; Kwade, A.; Herrmann, C. Exploring the Effect of Increased Energy Density on the Environmental Impacts of Traction Batteries: A Comparison of Energy Optimized Lithium-Ion and Lithium-Sulfur Batteries for Mobility Applications. *Energies* **2018**, *11*, 150. DOI: https://doi.org/10.3390/en11010150.
(56) Mayyas, A.; Steward, D.; Mann, M. The case for recycling: Overview and challenges in the material supply chain for automotive li-ion batteries. *Sustain. Mater. Technol.* **2019**, *19*, e00087. DOI: https://doi.org/10.1016/j.susmat.2018.e00087.
(57) Argue, C. *What can 6,000 electric vehicles tell us about EV battery health?* 2020. https://www.geotab.com/blog/ev-battery-health/ (accessed 25/08/2021).
(58) Shrivastava, R. *Electric Motor Life Expectancy: How Long do Electric Car Motor Last*. 2020. https://www.vehiclesuggest.com/electric-motor-life-expectancy/ (accessed 27/10/2021).
(59) Nakamoto, Y.; Nishijima, D.; Kagawa, S. The role of vehicle lifetime extensions of countries on global CO2 emissions. *Journal of Cleaner Production* **2019**, *207*, 1040-1046. DOI: https://doi.org/10.1016/j.jclepro.2018.10.054.
(60) USGS. *Cobalt [advanced release] - 2019 Minerals Yearbook (U.S. Geological Survey, 2020).* ; 2021.
(61) USGS. *Bauxite and Alumina [advanced release] - 2019 Minerals Yearbook (U.S. Geological Survey, 2020).* . 2021. (accessed.
(62) BGR. *Mining Conditions and Trading Networks in Artisanal Copper-Cobalt Supply Chains in the Democratic Republic of the Congo*; 2021.

(63) Sun, X.; Hao, H.; Liu, Z.; Zhao, F.; Song, J. Tracing global cobalt flow: 1995-2015. *Resour. Conserv. Recycl.* **2019**, *149*, 45-55. DOI: https://doi.org/10.1016/j.resconrec.2019.05.009.
(64) Berr, M.; Beloin-Saint-Pierre, D.; Hischier, R.; Hool, A.; Wäger, P. SPOTTER: Assessing supply disruption impacts along the supply chain within Life Cycle Sustainability Assessment. *Cleaner Logistics and Supply Chain* **2022**, *4*, 100063. DOI: https://doi.org/10.1016/j.clscn.2022.100063.
(65) World Bank. *Worldwide Governance Indicators*. 2019. https://info.worldbank.org/governance/wgi/#home (accessed 05/02/2019).
(66) Benoit Norris, C.; Bennema, M.; Norris, G. *THE SOCIAL HOTSPOTS DATABASE Supporting documentation Update 2019*; 2019.
(67) World Bank. *Trading across Borders*. 2020. https://www.doingbusiness.org/en/data/exploretopics/trading-across-borders (accessed 18/06/2020).
(68) Church, C.; Wuennenberg, L. *Sustainability and Second Life: The case for cobalt and lithium recycling*; 2019.
(69) The Aluminium Association. *Infinetely Recyclable - Circular Economy Solution*. 2021. https://www.aluminum.org/Recycling (accessed 20/02/2022).
(70) USGS. Cobalt [advanced release] - 2016 Minerals Yearbook (U.S. Geological Survey, 2020). . **2021**.
(71) USGS. Cobalt [advanced release] - 2017 Minerals Yearbook (U.S. Geological Survey, 2020). . **2021**.
(72) USGS. Cobalt [advanced release] - 2018 Minerals Yearbook (U.S. Geological Survey, 2020). . **2021**.
(73) Bach, V.; Finogenova, N.; Berger, M.; Winter, L.; Finkbeiner, M. Enhancing the assessment of critical resource use at the country level with the SCARCE method – Case study of Germany. *Resources Policy* **2017**, *53*, 283-299. DOI: https://doi.org/10.1016/j.resourpol.2017.07.003.
(74) Cimprich, A.; Young, S. B.; Helbig, C.; Gemechu, E. D.; Thorenz, A.; Tuma, A.; Sonnemann, G. Extension of geopolitical supply risk methodology: Characterization model applied to conventional and electric vehicles. *Journal of Cleaner Production* **2017**, *162*, 754-763. DOI: https://doi.org/10.1016/j.jclepro.2017.06.063.
(75) Cimprich, A.; Karim, K. S.; Young, S. B. Extending the geopolitical supply risk method: material "substitutability" indicators applied to electric vehicles and dental X-ray equipment. *The International Journal of Life Cycle Assessment* **2018**. DOI: https://doi.org/10.1007/s11367-017-1418-4.
(76) Lütkehaus, H.; Pade, C.; Oswald, M.; Brand, U.; Naegler, T.; Vogt, T. Measuring raw-material criticality of product systems through an economic product importance indicator: a case study of battery-electric vehicles. *The International Journal of Life Cycle Assessment* **2022**, *27* (1), 122-137. DOI: https://doi.org/10.1007/s11367-021-02002-z.
(77) Moss, R. L.; Tzimas, E.; Willis, P.; Arendorf, J.; Tercero Espinoza, L. Critical Metals in the Path towards the Decarbonization of the EU Energy Sector: Assessing Rare Metals as Supply-Chain Bottlenecks in Low-Carbon Energy Tehnologies. **2013**, (JRC - Institute for Energy and Transport). DOI: https://doi.org/10.2790/46338.
(78) Blagoeva, D. T.; Alves Dias, P.; Marmier, A.; Pavel, C. C. Assessment of potential bottlenecks along the materials supply chain for the future deployment of low-carbon energy and transport technologies in the EU. Wind power, photovoltaic and electric vehicles technologies, time frame: 2015-2030. **2016**. DOI: https://doi.org/10.2790/08169.
(79) Bobba, S.; Carrara, S.; Huisman, J.; Mathieux, F.; Pavel, C. *Critical Raw Materials for Strategic Technologies and Sectors in the EU – A Foresight Study*; 2020.
